# Distinct TP53 Mutation Types Exhibit Increased Sensitivity to Ferroptosis Independently of Changes in Iron Regulatory Protein Activity

**DOI:** 10.3390/ijms21186751

**Published:** 2020-09-15

**Authors:** Laurie R. Thompson, Thais G. Oliveira, Evan R. Hermann, Winyoo Chowanadisai, Stephen L. Clarke, McKale R. Montgomery

**Affiliations:** Department of Nutritional Sciences, Oklahoma State University, Stillwater, OK 74074, USA; laurie.thompson@okstate.edu (L.R.T.); thais.g.oliveira@okstate.edu (T.G.O.); evan.hermann@okstate.edu (E.R.H.); winyoo.chowanadisai@okstate.edu (W.C.); stephen.clarke@okstate.edu (S.L.C.)

**Keywords:** mutant TP53, ferroptosis, iron metabolism, iron regulatory proteins, cancer

## Abstract

The tumor suppressor gene *TP53* is the most commonly mutated gene in human cancer. In addition to loss of tumor suppressor functions, mutations in *TP53* promote cancer progression by altering cellular iron acquisition and metabolism. A newly identified role for TP53 in the coordination of iron homeostasis and cancer cell survival lies in the ability for TP53 to protect against ferroptosis, a form of iron-mediated cell death. The purpose of this study was to determine the extent to which TP53 mutation status affects the cellular response to ferroptosis induction. Using H1299 cells, which are null for TP53, we generated cell lines expressing either a tetracycline inducible wild-type (WT) TP53 gene, or a representative mutated TP53 gene from six exemplary “hotspot” mutations in the DNA binding domain (R273H, R248Q, R282W, R175H, G245S, and R249S). TP53 mutants (R273H, R248Q, R175H, G245S, and R249S) exhibited increased sensitivity ferroptosis compared to cells expressing WT TP53. As iron-mediated lipid peroxidation is critical for ferroptosis induction, we hypothesized that iron acquisition pathways would be upregulated in mutant TP53-expressing cells. However, only cells expressing the R248Q, R175H, and G245S TP53 mutation types exhibited statistically significant increases in spontaneous iron regulatory protein (IRP) RNA binding activity following ferroptosis activation. Moreover, changes in the expression of downstream IRP targets were inconsistent with the observed differences in sensitivity to ferroptosis. These findings reveal that canonical iron regulatory pathways are bypassed during ferroptotic cell death. These results also indicate that induction of ferroptosis may be an effective therapeutic approach for tumor cells expressing distinct TP53 mutation types.

## 1. Introduction

Iron is an essential, yet potentially toxic nutrient that can contribute to both the initiation and progression of cancer [1,2,3,4]. The tumor suppressor TP53 can protect against carcinogenesis by contributing to the regulation of cellular iron homeostasis [5,6,7]. Unfortunately, *TP53* is mutated in nearly half of all human cancers. Mutations in *TP53* can lead to both loss of tumor suppressive functions and the acquisition of oncogenic traits, but the influence on cellular iron homeostasis has yet to be fully described.

The cytosolic mRNA binding proteins, iron regulatory proteins (IRP1 and IRP2), function as key regulators of cellular iron homeostasis by coordinating iron uptake, storage, and utilization in accordance with cellular iron availability. When iron availability is limited, the binding of IRPs to iron responsive elements (IREs) within the 5′ untranslated region of mRNA such as ferritin heavy chain 1 (FTH1) results in translational inhibition [8]. Conversely, binding to IRE in the 3′ untranslated regions of mRNA, such transferrin receptor 1 (TFRC) promotes transcript stabilization. The net effect is to subsequently decrease iron storage and increase iron uptake, respectively. Under iron sufficient conditions, IRP2 is subject to proteasomal degradation, while IRP1 is regulated via the insertion of a Fe-S cluster, which prevents IRE binding [9].

In cancer, IRP signaling can be corrupted in an effort to acquire sufficient iron to support rapid cell proliferation. For example, IRP2 overexpression in breast cancer results in increased TFRC expression, decreased ferritin expression, and subsequently an increased labile iron pool [10]. Increased expression of TFRC also worsens clinical prognosis in patients with renal cell carcinoma [11]. As mentioned above, increased expression of TFRC is typically mediated by increased IRP mRNA binding activity, but overexpression of IRP1 was actually found to decrease tumor growth in vivo [12]. Thus, despite their similar roles in the maintenance of iron homeostasis, IRP1 and IRP2 exhibit opposing phenotypes in the reduction and promotion of tumor growth, respectively. Therefore, continued investigation into the roles IRPs play in cancer progression is warranted.

Ferroptosis is an iron-dependent form of programmed cell death with broad chemotherapeutic potential [13,14]. Driven by the iron-dependent accumulation of lipid reactive oxygen species (ROS), the import of iron by TFRC-mediated iron uptake is an essential component of ferroptotic cell death [15]. The increased expression of IRP2 and degradation of FTH1 have also been hypothesized to serve as critical contributors to ferroptosis induction, presumably as a means of increasing the redox-active labile iron pool [13,16]. Yet, the contribution of the IRE-IRP system to iron accumulation during ferroptotic cell death remains unclear.

Intriguingly, wild-type (WT) TP53 can contribute to both IRP regulation [5,7] and ferroptosis sensitivity in human cancer cells, though contradictory roles have been reported [17,18,19]. These mixed findings are likely attributable to the context-dependent TP53-mediated upregulation of Cyclin Dependent Kinase Inhibitor 1A (CDKN1A) expression that appears to be critical for suppression of ferroptosis [18]. Posttranslational modifications and/or genetic mutations within TP53 can render it unable to induce CDKN1A in some cell types resulting in increased ferroptosis sensitivity [17,20]. However, the mutants in these studies were acetylation defective mutants and not representative of the more common TP53 mutations within the DNA binding domain most often observed in human cancers. Moreover, the capacity for distinct TP53 subtypes to protect against ferroptosis by modulating IRP has yet to be fully investigated. 

The purpose of this study was to determine the extent to which ferroptosis induction influences iron metabolism and ferroptotic cell death in cells with distinct TP53 mutation types. We hypothesized that impaired IRP-mediated signaling pathways would render cells harboring *TP53* mutations more susceptible to ferroptotic cell death than cells expressing wild-type TP53. To test this hypothesis we assessed cell viability, IRP mRNA binding activity, and iron-mediated lipid peroxidation following erastin induction in isogenic cell lines expressing inducible versions of the six most commonly observed TP53 mutations in human cancers. We established that induction of distinct TP53 mutations alone significantly increased sensitivity to ferroptotic cell death. Contrary to our hypothesis, however, we show that mutant TP53-dependent differences in ferroptotic sensitivity are independent of IRP regulation, but rather, are likely multifactorial and dependent upon TP53 mutation type. These findings provide further evidence of the many phenotypic differences that can be observed between distinct TP53 mutation types, and illustrate the importance of distinguishing between subtypes when investigating mutant TP53-dependent outcomes.

## 2. Results

### 2.1. TP53 Mutation Status Influences Sensitivity to Erastin-Induced Ferroptotic Cell Death

**Figure 1:**
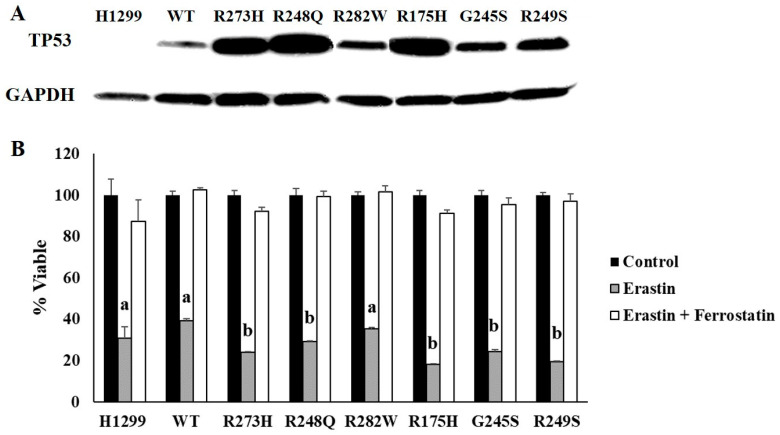
TP53 mutation status influences ferroptosis sensitivity. H1299 (TP53 null) cells were transfected with either a tetracycline inducible wild-type (WT) TP53 or a representative contact (273H, 248Q, 282W) or conformational (175H, 245S, 249S) mutant TP53. (**A**) Tetracycline-inducible expression of WT and mutant TP53 expression was confirmed by Western blot. Glyceraldehyde 3-phosphate dehydrogenase (GAPDH) was used as a loading control. (**B**) Cell viability measured following 24 h of treatment with DMSO (Control), 10 µM erastin, or 10 µM each of erastin and ferrostatin-1 (*n* = 4 per cell type and treatment group). Superscripts (^a,b^) denote statistical significance, *p* < 0.05. Treatments that share the same superscripts are not significantly different. Error bars indicate SEM.

To assess the influence of TP53 mutation status on ferroptosis sensitivity, H1299 cells expressing tetracycline-inducible wild-type (WT) or mutant *TP53* plasmids were generated, and their protein expression was validated via Western blot (Figure 1A). These mutations were selected because they represent the most commonly observed *TP53* mutation types in human cancers [21]. TP53 expression levels were variable between WT and mutant *TP53*-expressing subtypes, but we have previously demonstrated that even a low level induction of WT TP53 protein is sufficient to induce expression of the WT TP53 target, CDKN1A [22]. We also showed that CDKN1A expression was not increased by the induction of any of the mutant *TP53* subtypes examined [22]. This is important because upregulation of CDKN1A expression appears to be critical for suppression of ferroptosis [18]. Thus, we hypothesized that mutant *TP53*-expressing cells would be more sensitive to ferroptosis induction. TP53-dependent differences in ferroptosis sensitivity were determined by comparing differences in cell viability following treatment with erastin, a potent inducer of ferroptosis [13]. A two-factor ANOVA was conducted to compare the effects of TP53 mutation type on cell viability following treatment with erastin or erastin + ferrostatin confirmed a significant erastin effect, indicating that all cells examined were susceptible to at least some degree of ferroptotic cell death (Figure 1B). Additional post hoc analyses revealed that induction of R273H, R248Q, R175H, G245S, and R249S *TP53* mutations significantly increased sensitivity to ferroptosis induction, as evidenced by reduced cell viability compared to the WT TP53-expressing cells following erastin treatment (Figure 1B). The TP53 null (H1299) cells and cells expressing the R282W mutation type did not exhibit differences in sensitivity to erastin treatment compared to the cells expressing WT TP53 (Figure 1B). Importantly, co-treatment with ferrostatin, a potent ferroptosis inhibitor [23], was sufficient to rescue all cell types, demonstrating that the reduction in cell viability was indeed due to ferroptosis (Figure 1B). These results indicate that induction of distinct TP53 mutation type is sufficient to increase sensitivity of cells to iron-mediated ferroptotic cell death.

### 2.2. Ferroptosis Induction Differentially Affects IRP mRNA Binding Activity in Cells Expressing Distinct TP53 Mutation Types

**Figure 2:**
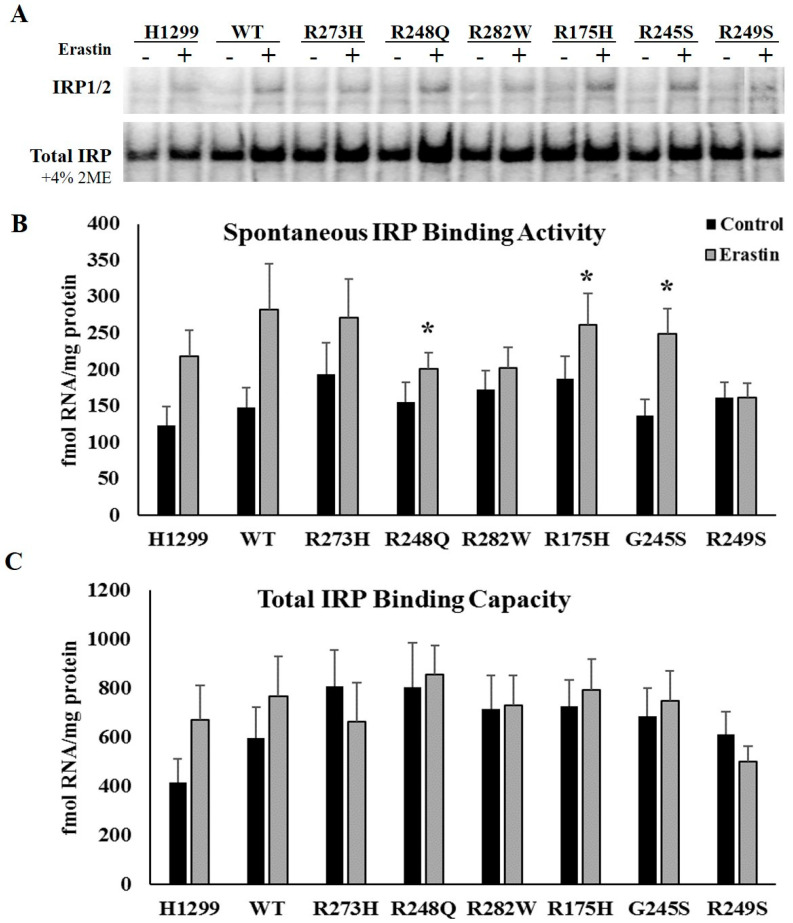
Erastin treatment increases iron regulatory protein (IRP)1/2 RNA binding activity in distinct mutant TP53-expressing subtypes. (**A**,**B**) Spontaneous IRP RNA binding was in tetracycline-induced WT and mutant TP53 (*n* = 3–4 per cell type and treatment) expressing subtypes following treatment with DMSO (control) or 10 µM erastin (Erastin) for 24 h. * Denotes statistical differences from respective control, *p* < 0.05. (**A**,**C**) Total IRP RNA binding capacity was measured by assaying the cells under the same conditions in the presences of 2-mercaptoethanol. Error bars indicate SEM.

We have previously established that induction of distinct *TP53* mutation types differentially influences IRP RNA binding activity and diminishes IRP responsiveness to changes in cellular iron availability [22]. As such, we hypothesized that mutant TP53-dependent differences in ferroptosis sensitivity might be influenced by mutant TP53-dependent differences in IRP RNA binding activity in response to erastin treatment. To examine the impact of ferroptosis induction on IRP function and expression, we quantitatively assessed spontaneous mRNA binding activity and total IRP mRNA binding capacity through an electrophoretic mobility assay (EMSA). Spontaneous IRP binding measures the amount of IRP1 and IRP2 in their active RNA binding forms under the specified control or treatment conditions. As iron-mediated lipid peroxidation is critical for ferroptosis induction [13], we hypothesized that spontaneous IRP mRNA binding activity would be increased in response to erastin treatment. However, despite upward trends in the TP53 null, WT TP53, and R273H TP53 mutants, only cells expressing the R248Q (*p* = 0.019), R175H (*p* = 0.015), and R245S (*p* = 0.027) TP53 mutation types exhibited statistically significant increases in spontaneous IRP RNA binding activity following erastin treatment (Figure 2A,B). Thus, erastin-mediated increases in IRP RNA binding activity cannot fully explain the increased sensitivity to ferroptotic cell death in all mutant TP53-expressing cell types.

The addition of 2-mercaptaethanol transiently prevents Fe-S assembly which allows for the measurement of total IRP1 protein levels, and thus reflects total IRP1 mRNA binding capacity [24]. This assay also informs us about the relative distribution of IRP1 in its mRNA binding form or its Fe-S cluster containing enzymatic form [25]. Total IRP1 mRNA binding activity was not affected by erastin treatment (Figure 2A,C). These results indicate that changes in spontaneous IRP binding activity were not the result of changes in the total pool of available IRP1 protein. Because human IRP1 and IRP2 do not separate during standard gel-shift analyses [26], the relative contributions of IRP1 versus IRP2 to IRE binding cannot be determined based on our current data. Therefore, the increased spontaneous IRP mRNA binding observed reflects either a removal of the Fe-S cluster from IRP1 RNA binding site, or an increased stability of IRP2 protein in response to erastin treatment.

### 2.3. Ferroptosis Induction and TP53 Status do not Influence Iron-Related mRNA Abundance

**Figure 3:**
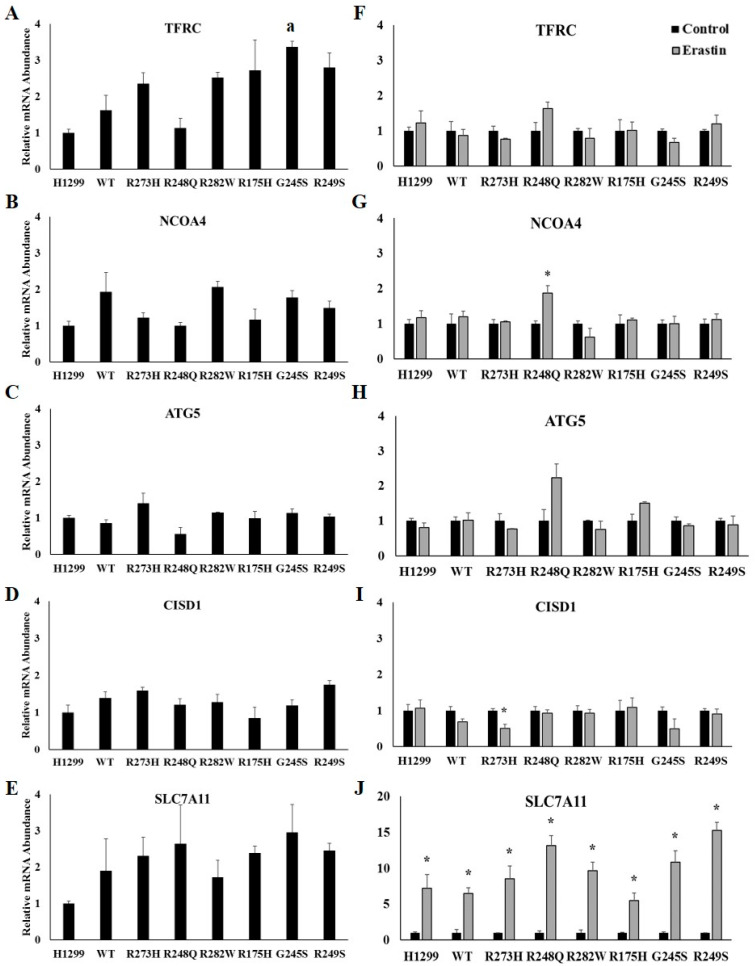
Iron-uptake and ferritinophagy-related gene expression are not affected by TP53 mutation status or erastin treatment. Relative mRNA expression of (**A**) transferrin receptor 1 (TFRC) (**B**) nuclear receptor coactivator 4 (*NCOA4*), (**C**) autophagy related 5 (*ATG5*), (**D**) CDGSG iron sulfur domain 1 (*CISD1*), and (**E**) solute carrier family 7 member 11 (*SLC7A11*) in TP53 null H1299 cells (H1299) or H1299 cells transfected with wild-type (WT) TP53, or the indicated TP53 mutant (*n* = 3 per cell type and treatment group). Relative mRNA expression of (**F**) *TFRC*, (**G**) *NCOA4*, (**H**) *ATG5*, (**I**) *CISD1*, and (**J**) *SLC7A11* in TP53 null H1299 cells (H1299) or H1299 cells transfected with wild-type (WT) TP53, or the indicated mutant TP53 following treatment with DMSO (control) or 10 µM erastin for 24 h. **^a^** Denotes statistical difference from H1299. * Denotes statistical differences from respective control, *p* < 0.05. Error bars indicate SEM.

The import of iron by TFRC and degradation of ferritin via ferritinophagy are critical components of ferroptotic cell death, with both IRP-dependent and -independent modes of regulation [15,16]. Therefore, we also investigated the influence of *TP53* mutation types and ferroptosis induction on the expression of *TFRC* and the ferritinophagy-related genes, nuclear receptor coactivator 4 (*NCOA4*), autophagy related 5 (*ATG5*), and CDGSG iron sulfur domain 1 (*CISD1*). First, we assessed the influence of induction of TP53 expression alone on iron-related mRNAs by normalizing changes in the relative abundance of the indicated mRNAs to that expressed in the TP53 null H1299 cell line (Figure 3A–E). A one-way between-subjects ANOVA comparing the effects of TP53 mutation type *TFRC* mRNA expression identified a significant effect at the *p* < 0.05 level for the eight cell types (F(7,16) = 5.302, *p* = 0.003). However, further post hoc analyses using Tukey HSD only revealed a significant difference between the TP53 null H1299 cells and the G245S-expressing mutants (Figure 3A). No other differences were observed following the induction of WT or mutant TP53 expression (Figure 3B–E). *TFRC* mRNA abundance was not affected by erastin treatment in any of the cell lines examined (Figure 3F). Thus, even though TFRC plays an essential role in ferroptosis [15], our data indicate that upregulation of TFRC is not necessary for ferroptosis to occur. *NCOA4* mRNA expression significantly increased following erastin treatment in H1299 cells expressing the R248Q TP53 mutant (Figure 3G). Conversely, *CISD1* mRNA abundance significantly decreased following erastin treatment in H1299 cells expressing the R273H TP53 mutant (Figure 3I). We also examined the mRNA abundance of Solute Carrier family 7 member 11 (*SLC7A11*), which is required for cystine import and has been shown to be upregulated following erastin treatment [18,23]. Importantly, there was a significant increase in *SLC7A11* mRNA abundance following erastin in each TP53-expressing subtype (Figure 3J). Thus, the observed increase in *SLC7A11* mRNA expression provides supporting evidence that ferroptosis was induced despite the lack of change in iron-related gene expression.

### 2.4. FTH1, but not TFRC Protein Expression, Is Altered by Erastin Treatment in Cells Expressing Distinct tp53 Mutation Types

**Figure 4:**
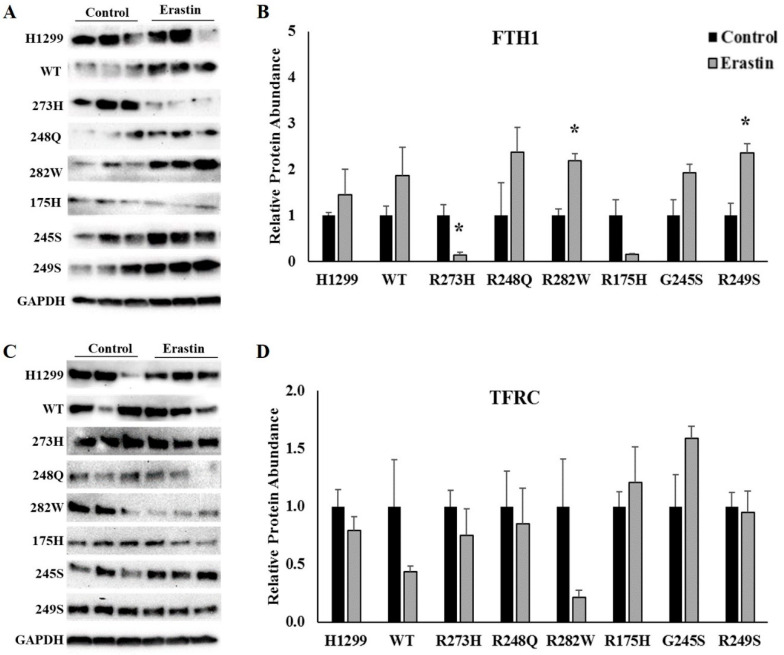
Erastin treatment differentially affects TFRC and ferritin heavy chain 1 (FTH1) protein expression in cells expressing distinct TP53 mutation types. Representative images and quantification of Western blots examining the expression of (**A**,**B**) transferrin receptor 1 (TFRC) and (**C**,**D**) ferritin heavy chain 1 (FTH1) in TP53 null H1299 cells (H1299) or H1299 cells transfected with wild-type (WT) TP53, or the indicated TP53 mutant following treatment with DMSO (control) or 10 µM erastin for 24 h (*n* = 3–4 per cell type and treatment). Relative expression was normalized using glyceraldehyde 3-phosphate dehydrogenase (GAPDH) as a loading control. * Denotes statistical differences from respective control, *p* < 0.05. Error bars indicate SEM.

While the IRP-mediated regulation of TFRC is modulated at the level of mRNA transcript stability, IRPs regulate FTH1 expression via translational inhibition [8]. Therefore, we used Western blots analyses to examine the effects of erastin treatment on the expression FTH1 in each of the cell lines described above. Unexpectedly, there was a significant increase in FTH1 protein expression following ferroptosis induction in R282W (*p* = 0.004), and R249S (*p* = 0.013) mutant TP53-expressing cells, but a significant decrease in FTH1 expression in the R273H-expressing mutants (*p* = 0.020) (Figure 4A). It is worth noting however, that IRP RNA binding activity was not significantly altered in any of the cell types in which FTH1 expression was changed. Independently of iron and IRPs, FTH1 can also be transcriptionally activated in response to oxidative stress [27,28]. As increased lipid peroxidation is a hallmark of ferroptosis, the increase in FTH1 expression in the R282W and R249S TP53 mutants could reflect an oxidative stress response. Given the importance of TFRC-mediated iron uptake to ferroptosis induction [15], and previous reports of post-translational regulation of TFRC expression independently of IRP [29,30,31], we also examined the protein expression of TFRC following erastin treatment. Consistent with our mRNA data however, there were no significant changes in TFRC protein expression following ferroptosis induction in any of the cell lines examined (Figure 4B).

### 2.5. TP53 Mutation Status Influences Basal Levels of Lipid Peroxidation and Subsequent Responsiveness to Erastin Treatment

**Figure 5:**
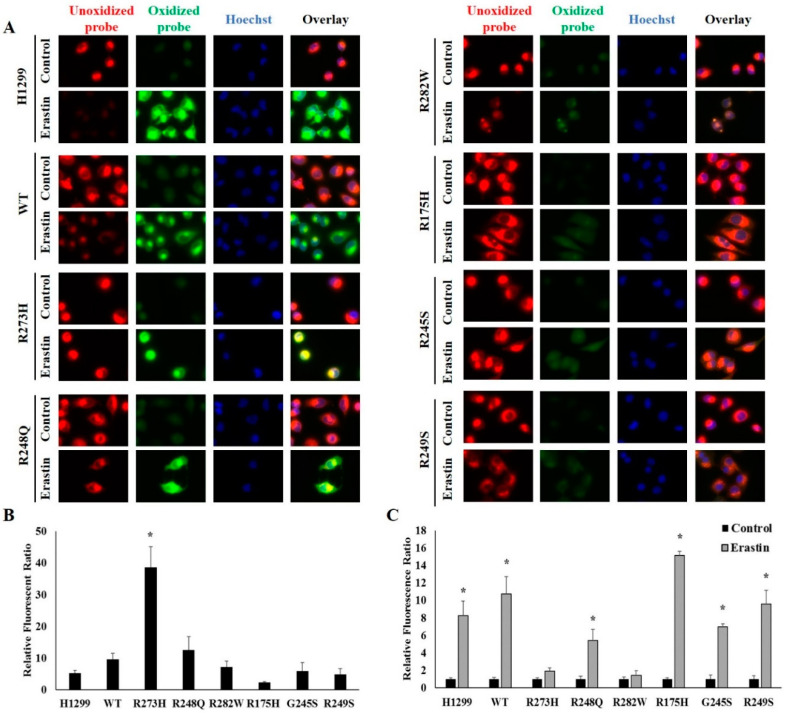
Mutant TP53 expression influences basal and erastin-induced levels of lipid peroxidation. (**A**) Unoxidized and oxidized C11-BODIPY in tetracycline-induced WT and mutant TP53-expressing following treatment with DMSO (control) or 10 µM erastin (Erastin) for 24 (*n* = 3–4 per cell type and treatment). (**B**,**C**) Changes in relative amounts of oxidized probe were quantified using ImageJ software following normalization to Hoechst nuclear staining to account for differences in cell number. (**B**) Student’s *t*-tests were used to identify statistically significant erastin responses relative to their controls within a given cell type. Relative fluorescence ratios for erastin-treated cells are shown normalized to the relative fluorescence ratios of their respective controls. * Denotes significant difference from control. (**C**) A one-way between-subjects ANOVA identified a significant effect of TP53 mutation type on basal oxidized probe at the *p* < 0.05 level for the 8 cell types (F (7,16) = 13.563, *p* < 0.001). * Post hoc comparisons using the Tukey LSD test indicated that basal levels of lipid peroxidation in the R273H-expressing mutants was significantly different from the WT TP53-expressing cells (*p* < 0.001). All images were acquired using a 100X objective under immersion oil. Error bars indicate SEM.

Reduced expression of functional WT TP53 has previously been demonstrated to amplify erastin-mediated lipid peroxidation [32]. Thus, we next investigated whether mutant TP53-dependent differences in erastin sensitivity were driven by variations in levels of lipid peroxidation. To do so, cells were incubated with a fluorescent lipid peroxidation sensor and differences in the level of oxidized probe in cells following tetracycline induction of WT and mutant TP53 expression. Intriguingly, R273H mutant TP53-expressing cells displayed higher levels of basal lipid peroxidation than any of there TP53 subtypes examined (Figure 5A,B). We then assessed the influence of ferroptosis on lipid peroxidation by measuring changes in the amount of oxidize probe following 24 h of treatment with erastin or DMSO (control). To account for differences in basal levels of lipid peroxidation, the changes in relative fluorescence ratio in the erastin-treated cells were normalized to that of their respective controls. Erastin treatment significantly increased lipid peroxidation in all cells tested, except for those cells expressing the R282W and R273H TP53 mutation types (Figure 5A,C). The lack of erastin-mediated lipid peroxidation in the R273H-expressing TP53 mutants may be due to high basal levels lipid peroxidation, which perhaps represent a maximal threshold. 

### 2.6. Human Cancer Cells Expressing Distinct Endogenous TP53 Mutation Types Exhibit Increased Ferroptotic Sensitivity

**Figure 6:**
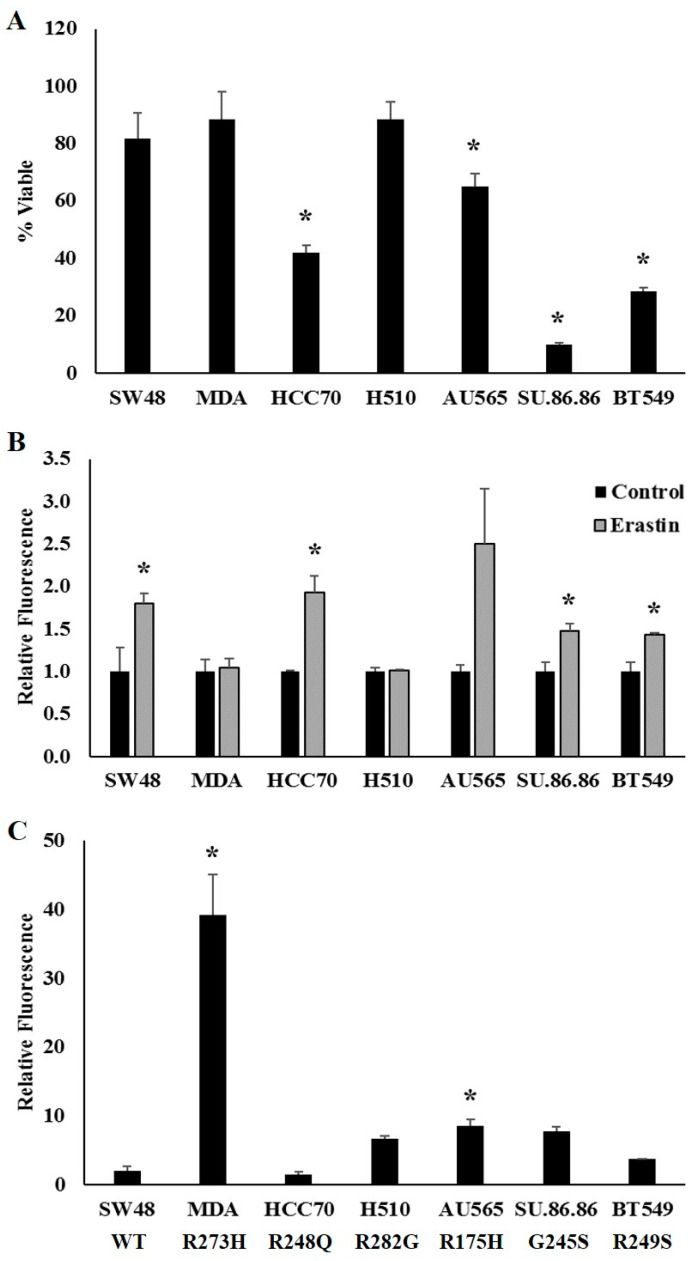
Cells expressing distinct TP53 mutation types exhibit increased ferroptotic sensitivity. (**A**) Cell viability measured following 24 h of treatment with 20 µM erastin in cells expressing endogenous WT TP53, or one of the TP53 mutation types indicated. * Denotes difference from WT TP53, *p* < 0.05. (**B**,**C**) Lipid peroxidation was assessed by measuring relative changes in fluorescence of unoxidized versus oxidized C11-BODIPY probe following treatment with DMSO (control) or 20 µM erastin (Erastin) for 24 h (*n* = 3–4 per cell type and treatment). (**B**,**C**) Changes in relative amounts of oxidized probe were quantified according to cell viability. (**B**) Student’s *t*-tests were used to identify statistically significant erastin responses relative to their controls within a given cell type. * Denotes significant difference from control. (**C**) A one-way between-subjects ANOVA identified a significant effect of TP53 mutation type on basal oxidized probe at the *p* < 0.05 level for the 8 cell types (F (6,14) = 24.954, *p* = 0.000). * Post hoc comparisons using the Tukey LSD test indicated that basal levels of lipid peroxidation in the R273H- (*p* = 0.000) and R175H- (*p* = 0.041) expressing mutants were significantly different from the WT TP53-expressing cells. Error bars indicate SEM.

We next sought to determine whether cells derived from tumors expressing endogenous TP53 mutations also displayed differences in sensitivity to ferroptosis induction. To assess the influence of distinct endogenous TP53 mutation types, we examined a panel of cell lines expressing endogenous WT TP53 (SW48), or similar representative hotspot TP53 mutations that were used in the experiments described above: R273H (MDA-MB-468), R248Q (HCC70), R282W (NCI-H510), R175H (AU565), G245S (SU.86.86), and R249S (BT549). One exception was made as NCI-H510 cells express an R282G instead of an R282W mutation, but an endogenously expressing R282W mutant was not commercially available. These cell lines were, in general, more resistant to ferroptosis than the H1299 cell line, and thus differences in ferroptotic sensitivity were assessed by treating cells with 20 µM of erastin for 24 h (instead of 10 µM). However, in agreement with our findings in the exogenously expressing TP53 mutant cell lines, cells expressing endogenous R248Q, R175H, G245S, and R249S TP53 mutation types exhibited increased sensitivity to ferroptosis induction compared to the cells expressing endogenous WT TP53 (Figure 6A). Cells endogenously expressing the R282G mutation type exhibited a similar erastin response to the WT TP53-expressing cells, as did the exogenously expressing R282W mutants. In contrast to the findings in the exogenously expressing R273H mutants, the endogenously expressing R273H mutants were no more sensitive to erastin treatment than their WT TP53-expressing controls.

Given the similarities in erastin-mediated changes cell in viability compared to the cells expressing exogenous TP53, we also investigated whether endogenous mutant TP53-dependent differences in erastin sensitivity might drive discrepancies in levels of lipid peroxidation. Following erastin treatment, the endogenous WT and mutant TP53-expressing cell lines were incubated with the same fluorescent lipid peroxidation sensor used above and differences in the levels of oxidized probe were measured using a plate reader with fluorescent capabilities. In line with the cells expressing exogenous TP53, cells expressing endogenous WT TP53, as well as R248Q, G245S, and R249S TP53 mutants displayed a significant increase in lipid peroxidation in response to erastin treatment (Figure 6B). The increase in lipid peroxidation following erastin treatment in the cells expressing the endogenous R175H mutant did not reach statistical significance. However, also in agreement with the H1299 cells expressing the exogenous R273H and R282W TP53 mutations, the endogenously expressing R273H and R282G mutant cell lines did not exhibit an increase in lipid peroxidation in response to erastin treatment. Moreover, as with the exogenously expressing R273H mutants, the cells expressing an endogenous R273H mutant also displayed remarkably high levels of basal lipid peroxidation compared to the other cell lines tested (Figure 6C).

## 3. Discussion

Cancer cells are extravagant users of iron, and as such, much effort has been devoted to taking advantage of cancers cells’ “iron addiction” by restricting iron availability (reviewed in [33,34,35]). However, these approaches are confounded by the essential nature of iron for noncancerous cells as well. Ferroptosis has been described as a novel approach to exploiting the toxic nature of iron to promote programmed cell death, but the toxic potential of iron for all cell types must be considered. Thus, we sought to investigate the potential to exploit the frequency of *TP53* mutations in human cancer to more favorably induced iron-mediated cell death in tumors harboring these mutation types.

Though the *TP53* mutation spectrum is vast, the majority of mutations occur within the DNA binding domain. *TP53* mutation types can generally be classified as either DNA contact mutants or conformational-type mutants depending on whether the mutation affects contact with target DNA or disrupts the structure of the TP53 protein, respectively. Such distinctions are important because they can dramatically influence phenotypic effects. In this study, we utilized cell lines expressing the most common examples of TP53 contact (R248Q, R273H and R282W) and conformational (R175H, G245S, and R249S) mutants [21]. Of the three contact mutation types investigated in the current study, only the R273H and R248Q TP53 exogenous mutant-expressing cell lines were more sensitive to ferroptotic cell death than WT TP53-expressing cells. However, the R282W mutants responded similarly to the WT TP53-expressing cells. On the other hand, the three conformation mutation types examined in this study (175H, G245S, and R249S) were all consistently more sensitive to ferroptosis induction than WT TP53-expressing cells. Importantly, we were then able to validate these findings in cells expressing endogenous WT and mutant TP53 as well. In the cells expressing endogenous TP53 mutation types, only one of the contact mutants (R248Q) was more sensitive to ferroptotic cell death, while all three conformational mutants examined exhibited increased ferroptotic sensitivity. Further investigation is needed to determine whether conformational-type TP53 mutants are uniformly more susceptible to iron-mediated cell death.

To investigate the extent to which the increase in IRP RNA binding activity was indeed responsible for the increase in ferroptotic sensitivity in the R248Q-, R175H-, and G245S-expressing mutants, we examined the expression of IRP targets, TFRC and FTH1. As transferrin-bound iron uptake via TFRC is essential for ferroptosis [15], we hypothesized that increased IRP RNA binding activity in these cells would result in increased TFRC expression, thereby increasing free iron availability and promoting ferroptotic cell death. However, we did not observe any changes in *TFRC* mRNA or protein expression following erastin treatment in any of the cell lines examined. Previous research investigating the regulation of TFRC upon ferroptosis induction has produced conflicting results. Wang et al. reported reduced TFRC expression following erastin treatment [36]. Such results are consistent with an appropriate cellular response, wherein IRPs sense a relative cellular iron overload and decrease mRNA binding to reduce TFRC expression and subsequently cellular iron uptake. Conversely, Alvarez et al. reported an increase in TFRC expression following erastin treatment [37]. The authors speculated that the increase in TFRC expression resulted from a decrease in Fe-S biogenesis/stability and a subsequent increase in IRP1 RNA binding activity. Nonetheless, neither IRP1 nor IRP2 expression or activity were assessed in either of these studies. The inconsistency between observations of TFRC responsiveness between these studies, and ours, is likely due to the differences in experimental models. Nonetheless, these findings demonstrate that iron availability during ferroptosis can be mediated via IRP-independent mechanisms.

Similarly, ferritin expression was not changed in any of the cell lines for which an increase in IRP RNA binding activity was observed. Further confounding our initial hypotheses, ferritin expression was decreased in the R273H-expressing mutants, but increased in the R249S and R282W mutants. The degradation of ferritin via ferritinophagy is an IRP-independent mode of ferritin regulation with an established role in ferroptotic cell death [16,38]. Therefore, we investigated the potential for ferritinophagy to contribute to ferritin regulation and ferroptosis sensitivity in mutant TP53-expressing cells. We did not detect differences in the expression of any of the ferritinophagy-related genes following erastin treatment in any of the cell lines examined. Thus, the mechanisms contributing to increased ferroptotic sensitivity in mutant *TP53*-expressing cells are complex and may not be consistent between distinct TP53 mutation types. Further investigation is warranted because increased ferritin expression in the R282W mutant *TP53*-expressing cells, which were less sensitive to erastin treatment, may be indicative of a unique protective mechanism against ferroptosis in this particular *TP53* mutation type. Future studies should investigate the IRP-independent modes of iron regulation in mutant *TP53*-expressing cells.

Independently of how iron becomes available, it is the peroxidation of lipids by free iron within the cell that ultimately leads to ferroptotic cell death [13,39]. Therefore, we examined whether mutant TP53-dependent changes in oxidized lipid accumulation in response to ferroptosis induction could explain the observed differences ferroptosis sensitivity. Intriguingly, though all cells succumbed to some level of ferroptotic cell death, erastin treatment did not significantly increase lipid peroxidation in the R273H and R282W *TP53*-expressing mutants. This was also true in the cells expressing endogenous R273H and R282W *TP53* mutation types.

To further investigate why lipid peroxidation did not increase in the R273H and R282W *TP53* mutant-expressing cell lines in response to erastin treatment, we analyzed basal levels of lipid peroxidation following induction of *TP53* expression alone. Both the exogenous and endogenous R273H *TP53*-expressing mutants displayed significantly higher levels of basal lipid peroxidation compared to the other *TP53* subtypes tested. These results are in agreement with previous work demonstrating that H1299 cells expressing an R273H mutation have impaired glutamate release and diminished baseline glutathione levels [40]. Future work should investigate why the endogenously expressing R273H *TP53* mutants were more resistant to ferroptosis. On the contrary, despite a lack of erastin responsiveness, in either the exogenous R282W or endogenous R282G *TP53*-expressing mutants, basal levels of lipid peroxidation were similar to WT in both groups. These findings indicate that R282W and R282G *TP53* mutations may have more capacity to combat lipid peroxidation than other *TP53* mutation types through a yet undefined mechanism. Future studies should examine differences in antioxidative capacity between cells expressing distinct mutant *TP53* types.

A recognized limitation of the current study was the primary use of a single isogenic cell line (H1299). However, by removing the variability associated with differences in genetic backgrounds and tumor sites, the use of a single cell line is a significant strength as well. Thus, the results can be attributed solely to differences in *TP53* mutation type. Our findings also highlight the importance of distinguishing between mutant *TP53* subtypes when investigating phenotypic effects. Previous work has shown that substitution of a different amino acid, even at the same position, can substantially alter phenotypic outcomes [41]. Herein, we show that induction of five of the six most common *TP53* mutations observed in human cancers are increases in sensitivity to ferroptosis in the human lung adenocarcinoma H1299 cell line. We further support these findings by validating our results in cell lines derived from a diverse set of malignancies. Future studies should seek to determine whether ferroptosis induction will be a viable approach for targeting tumors expressing distinct *TP53* mutation types.

## 4. Materials and Methods

### 4.1. Cell Culture and Creation of Stable Mutant TP53-Expressing Cell Lines

Cells containing endogenous WT TP53 (SW48) or an endogenous R273H (MDA-MB-468), R248Q (HCC70), R282G (NCI-H510), R175H (AU565), G245S (SU.86.86), or R249S (BT549) TP53 mutation were also obtained from the American Type Culture collection (ATCC). All cell lines were maintained according to the instructions provided on the provider’s website. TP53 null, H1299 cells, were also obtained from ATCC and maintained in RPMI-40 (Corning) containing 10% tetracycline-free FBS (Atlanta Biologicals, Norcrsoss, GA, USA) and 100 IU/mL penicillin and 100 (µg/mL) streptomycin in a temperature and humidity-controlled incubator. Tetracycline-inducible expression plasmids (pcDNA5/TO; ThermoFisher; Waltham, MA, USA) containing either wild-type TP53, or a representative “hotspot” TP53 mutant (R273H, R248Q, R282W, R175H, G245S, R245G, or R249S) were generated and validated by Sanger sequencing (See Supplementary Files 1–13) using a custom cloning service GenScript (Piscatawy, MJ, USA). To obtain stable, tetracycline-inducible TP53-expressing cell lines, H1299 cells were co-transfected with a Tet-repressor plasmid (pcDNA/TR; ThermoFisher; Waltham, MA, USA) and one of the tetracycline-inducible plasmids mentioned above using lipofectamine 3000 reagent (ThermoFisher; Waltham, MA, USA) followed by selection with 6 µg/mL blasticidin and 600 µg/mL hygromycin. In total, eight cell lines were generated. H1299 cells expressing only the pcDNA6/TR and empty pcDNA5/TO vectors (H1299) were used as TP53 null control cells. The seven other cell lines expressed both pcDNA6/TR and either a wild-type TP53 gene (WT), or one of the representative TP53 hotspot mutations mention above. These mutations were selected because they represent the most common examples of p53 contact (R248Q, R273H and R282W) and conformational (R175H, G245S, and R249S) mutation types. TP53 expression was induced by supplementing the media with 10 µg/mL tetracycline for 24 h. The efficacy of tetracycline-dependent induction of TP53 was validated by Western blot.

### 4.2. Ferroptosis Induction and Cell Viability Assessment

H1299 cells expressing exogenous WT or mutant TP53 were seeded at 4 × 10^3^ cells per well in a 96-well plate and allowed to grow for 24 h before treatment with 5 µM erastin, or a vehicle control (DMSO) along with 10 µg tetracycline for 24 h. Co-treatment with 10 µM ferrostatin-1, a ferroptosis inhibitor, was used as a negative control. Cell viability was assessed using PrestoBlue reagent (Thermo Fisher Scientific, Waltham, MA, USA) by adding 10 µL of PrestoBlue reagent to each followed by incubation at 37 °C for 20 min. Differences in fluorescence absorbance were measured using a Biotek Synergy HT (Biotek, Winooski, VT, USA) plate reader. Each assay included four technical replicates and was repeated at least three times. Differences in cell viability were determined by normalizing reductions in fluorescent absorbance relative to the vehicle control group for each cell line. To adjust for differences in size in the cells expressing endogenous WT or mutant TP53, cells were plated such that they were ~70% confluent on the day of treatment. Cells were treated with a vehicle control (DMSO) or 20 µM erastin for 24 h, and cell viability was assessed using PrestoBlue reagent as described above.

### 4.3. Electrophoretic Mobility Shift Assays

Spontaneous and total IRP RNA binding activity was determined by EMSA as previously described [42]. Briefly, cells were treated with 10 µM erastin and 10 µg tetracycline for 24 h and harvested from 25-cm plates at 90% confluency. Cytosolic protein was collected by lysing cells in two-volumes cytosol buffer (1 M HEPES 10 mM, 10 mM KCl, 1%, 0.1 mM EGTA, 0.1 mM EDTA) supplemented with 1 mM citrate, 1 mM DTT, 0.1 M PMSF, and 100X Halt Protease and Phosphatase inhibitor cocktail (ThermoFisher; Waltham, MA, USA). After 15 min of incubation on ice, NP40 was added to a final volume of 1%, and the samples were vortexed for 10 s. Following centrifugation at 12,000× *g* for 10 min at 4 °C, the supernatant (cytosol) was collected, concentration was determined by bicinchoninic acid (BCA) assay (Pierce, ThermoFisher; Waltham, MA, USA), and samples were store in liquid nitrogen until use. Spontaneous IRP RNA binding activities were assessed by incubating 10 µg cytosolic protein with saturating levels (1 nM) of [^32^P]-labeled RNA from the rat L-ferritin IRE. Total IRP1 RNA binding activity was measured by adding 10 µg of cytosolic protein to saturating levels of [^32^P]-labeled RNA in the presence of 4% 2-mercaptoethanol. RNA binding activity was quantified using Optiquant Acquisition and Analysis software (Packard Bioscience, Meriden, CT, USA) (Supplementary Figure S1).

### 4.4. Western Blots

Following their respective treatments, cells were collected into 1.5-mL microcentrifuge tubes and washed with 1X PBS. Total protein was isolated by lysing cells in radioimmunoprecipitation (RIPA) buffer (50 mM Tris-HCL, pH 8.0, 1% NP-40, 0.5% Na-deoxycholate, 0.1% SDS, 2 mM EDTA, 150 mM NaCl) supplemented with Halt Protease and Phosphatase Inhibitor Cocktail (ThermoFisher; Waltham, MA, USA), 1 mM DTT, 1 mM citrate, 0.1 mM phenylmethylsulfonyl fluoride, and 10 µM MG-132. Following centrifugation at 14,000× *g* for 20 min, the supernatant was collected, and protein concentration was determined by BCA assay.

For Western blot analysis, 30 μg of total protein was solubilized in 2× Laemmli sample buffer (4% SDS, 20% glycerol. 0.0004% bromophenol blue, 0.125 M Tris-HCl, pH 6.8, 10% 2-mercaptoethanol) then boiled at 95 °C for 4 min. Proteins were then separated using a (Bio-Rad Mini-PROTEAN^®^ TGX™, Hercules, CA, USA) 4–20% gradient SDS gel at 150 volts for 60 min before being transferred to a PVDF membrane. Equal transfer was confirmed with Ponceau-S staining before blocking of the membrane for 60 min in 5% non-fat dried milk in 1× TBS and 0.01% Tween-2) (5% NDFM-T) at room temperature. The membranes were incubated with primary antibodies diluted in the 5% NFDM-T at the following concentrations: CD71 (sc-9099) (H-300) (Santa Cruz Biotechnologies, Santa Cruz, CA, USA) at 1:100 dilution, CD71 (D7S5Z) #13208 (Cell Signaling Technology, Danvers, MA, USA) at 1:1000 dilution, FTH1 (D1D4) #4393S (Cell Signaling Technology, MA, USA) at 1:1000 dilution and loading control glyceraldehyde 3-phosphate dehydrogenase (GAPDH) (sc-47724) (0411) (Santa Cruz Biotechnologies, Santa Cruz, CA, USA) at 1:1000 dilution.

The membranes were then washed three times in 5% NFDM-T to remove any residual primary antibody. The membranes were then incubated in the following secondary antibodies (diluted in the 5% non-fat dry milk—TBS Blotto): Anti-Rabbit IgG, HRP-linked Antibody #7074P2 (Cell Signaling Technology, MA, USA) and Anti-Mouse IgG, HRP-linked Antibody #7076P2 (Cell Signaling Technology, MA, USA) both at 1: 10,000 for one hour at 4 °C. Membranes were then washed three times using the 5% NFDM-T for five minutes each time, then washed two times with 1X TBS. Protein bands were then visualized using (SuperSignal™ West Pico PLUS Chemiluminescent Substrate kit; Pierce, WA, USA) following the manufacturer’s protocol (Supplementary Figure S2). Chemiluminescence signal was then viewed using a FluorChem R ProteinSimple fluorescence imaging system (R&D Systems; Minneapolis, MN, USA) and analyzed using ImageJ software [43].

### 4.5. RNA Isolation and Real-Time qPCR

To assess changes in mRNA abundance following induction of ferroptosis, cells were seeded in a 6-well plate at 1 × 10^5^ cells/well and incubated for 24 h before co-treatment with 10 µM erastin and 10 µg tetracycline for 24 h. Total RNA was isolated using TRIzol reagent (ThermoFisher; Waltham, MA, USA). RNA purity and integrity were confirmed by Nanodrop (ThermoFisher) and agarose gel electrophoresis, respectively, before reverse-transcription using SuperScript II (Invitrogen, Carlsbad, CA, USA). The relative abundance of *TFRC*, *NCOA4*, *ATG5*, *CISD1*, *SLC7A11*, and ferroportin (Supplementary Figure S3) were determined by real-time qPCR using SYBR green chemistry on an ABI 7900HT Real-Time PCR system (ThermoFisher; Waltham, MA, USA) and normalized relative to peptidylprolyl isomerase B (*PPIB*) abundance using the 2^−ΔΔCt^ method (User Bulletin no. 2, Applied Biosystems). Primer sequences for each mRNA of interest were obtained from previously published sources: PPIB [22], TFRC [44], NCOA4 [45], ATG5 [46], CISD1 [47], and SLC7A11 [48].

### 4.6. Lipid Peroxidation Assays

To assay TP53-dependent differences in lipid peroxidation following erastin treatment in the tetracycline-inducible cells, all cell lines were plated in an 8-well chamber slide (Ibidi, Martinsried, Germany) at 10,000 cells/well. Cells were treated with 10 µM erastin and 10 µg tetracycline for 24 h. The cells were then washed with 1X Hank’s balanced salt solution (HBSS) before incubation with 5 µM BODIPY 581/591 C11 and Hoechst stain (1:1000) (ThermoFisher; Waltham, MA, USA) for 10 min at 37 °C. Then, the mixture of 1X HBSS and reagent was aspirated and 1× HBSS was added to the cells. Cells were imaged using the BZ-X700 Life Science Microscope (Keyence, Osaka, Japan) with a 100× objective lens under immersion oil. Low photobleach settings and exposure times were held consistent through the imaging process. Increased lipid peroxidation was shown with oxidation of the polyunsaturated butadienyl portion of the dye resulting in a change from 590 to 510 nm excitation. ImageJ software was used to measure differences in the ratio of red and green fluorescence intensities, which were then normalized to cell number by counting the number of Hoechst stained nuclei [43]. To measure lipid peroxidation in the cell lines expressing endogenous WT or mutant TP53, cells were plated into black-wall, clear bottom 96-well plates such that they were 70% confluent on the day of treatment. Cells were treated with DMSO as a vehicle control or with 20 µM erastin for 24 h. On the day of measurement, cells were washed once with HBSS, incubated with 5 µM BODIPY 581/591 C11 (ThermoFisher; Waltham, MA, USA) at 37 °C for 20 min, and then washed once more with HBSS. Fluorescence was read at 488 nm_excitation_/510 nm_emission_ and 581 nm_excitation_/591 nm_emission_ wavelengths using Biotek Synergy H1 (Biotek, Winooski, VT, USA) plate reader. The fluorescence ratio, 591 nm_[reduction]_/510 nm_[oxidation]_, was calculated and normalized to cell viability, which was assessed in parallel plates using PrestoBlue reagent (Thermo Fisher Scientific, Waltham, MA, USA).

### 4.7. Statistical Analysis

One-way ANOVA was used to assess differences in treatment responses between cell lines following induction of TP53 expression. A two-factor mixed design ANOVA was performed to assess differences in responsiveness to treatment between cells. When statistically significant effects were identified by ANOVA, post hoc analyses were performed to make pairwise comparisons using the Tukey HSD method. Student’s *t*-tests were used to identify statistically significant treatment responses relative to their controls within a given cell type. Differences were considered statistically significant at the 95% confidence level (alpha = 0.05). Descriptive statistics were calculated for all variables and include mean ± SEM. All experiments were repeated 3 times, with *n* = 3 per group. All tests were performed using SPSS v23.0 software (IBM-SPSS; Chicago, IL, USA).

## 5. Conclusions

To date, the targeting of mutant TP53 has primarily focused on restoring its wild-type activity, or promoting its degradation, while iron chelation has been a primary emphasis for the development of iron-based chemotherapy. In this study, we have established that induction of distinct TP53 mutation types increases sensitivity to ferroptotic cell death. These findings are novel because they describe an approach that would allow for the exploitation of mutant TP53 expression to more favorably induce iron-mediated cell death via the activation of ferroptosis. We have also demonstrated that the IRP response to erastin treatment is dependent upon TP53 mutation type and is not essential for ferroptosis induction. Our findings are strengthened by examining the most prevalent TP53 mutations that represent exemplary models of both contact and conformational mutants. As these mutation types represent some of the most prevalent TP53 mutations in human tumors, these findings are relevant to a variety of clinically important cancers.

## Supplementary Materials

The following are available online at https://www.mdpi.com/1422-0067/21/18/6751/s1. Supplementary Files 1–13; Figure S1. Raw Gel Shift Images for Manuscript; Figure S2. Raw Western Blot Images for Manuscript; Figure S3. Ferroportin (SLC40A1) mRNA expression.

## References

[1] Campbell J.A. (1940). Effects of Precipitated Silica and of Iron Oxide on the Incidence of Primary Lung Tumours in Mice. Br. Med. J..

[2] Hann H.W., Stahlhut M.W., Blumberg B.S. (1988). Iron nutrition and tumor growth: Decreased tumor growth in iron-deficient mice. Cancer Res..

[3] Hann H.W., Stahlhut M.W., Menduke H. (1991). Iron enhances tumor growth. Observation on spontaneous mammary tumors in mice. Cancer.

[4] Richmond H.G. (1959). Induction of sarcoma in the rat by iron-dextran complex. Br. Med. J..

[5] Funauchi Y., Tanikawa C., Yi Lo P.H., Mori J., Daigo Y., Takano A., Miyagi Y., Okawa A., Nakamura Y., Matsuda K. (2015). Regulation of iron homeostasis by the p53-ISCU pathway. Sci. Rep..

[6] Shen J., Sheng X., Chang Z., Wu Q., Wang S., Xuan Z., Li D., Wu Y., Shang Y., Kong X. (2014). Iron metabolism regulates p53 signaling through direct heme-p53 interaction and modulation of p53 localization, stability, and function. Cell Rep..

[7] Zhang F., Wang W., Tsuji Y., Torti S.V., Torti F.M. (2008). Post-transcriptional modulation of iron homeostasis during p53-dependent growth arrest. J. Biol. Chem..

[8] Anderson C.P., Shen M., Eisenstein R.S., Leibold E.A. (2012). Mammalian iron metabolism and its control by iron regulatory proteins. Biochim. Biophys. Acta.

[9] Johnson N.B., Deck K.M., Nizzi C.P., Eisenstein R.S. (2017). A synergistic role of IRP1 and FBXL5 proteins in coordinating iron metabolism during cell proliferation. J. Biol. Chem..

[10] Wang W., Deng Z., Hatcher H., Miller L.D., Di X., Tesfay L., Sui G., D’Agostino R.B., Torti F.M., Torti S.V. (2014). IRP2 regulates breast tumor growth. Cancer Res..

[11] Greene C.J., Attwood K., Sharma N.J., Gross K.W., Smith G.J., Xu B., Kauffman E.C. (2017). Transferrin receptor 1 upregulation in primary tumor and downregulation in benign kidney is associated with progression and mortality in renal cell carcinoma patients. Oncotarget.

[12] Chen G., Fillebeen C., Wang J., Pantopoulos K. (2007). Overexpression of iron regulatory protein 1 suppresses growth of tumor xenografts. Carcinogenesis.

[13] Dixon S.J., Lemberg K.M., Lamprecht M.R., Skouta R., Zaitsev E.M., Gleason C.E., Patel D.N., Bauer A.J., Cantley A.M., Yang W.S. (2012). Ferroptosis: An iron-dependent form of nonapoptotic cell death. Cell.

[14] Lu B., Chen X.B., Ying M.D., He Q.J., Cao J., Yang B. (2017). The Role of Ferroptosis in Cancer Development and Treatment Response. Front. Pharmacol..

[15] Gao M., Monian P., Quadri N., Ramasamy R., Jiang X. (2015). Glutaminolysis and Transferrin Regulate Ferroptosis. Mol. Cell.

[16] Hou W., Xie Y., Song X., Sun X., Lotze M.T., Zeh H.J., Kang R., Tang D. (2016). Autophagy promotes ferroptosis by degradation of ferritin. Autophagy.

[17] Jiang L., Kon N., Li T., Wang S.J., Su T., Hibshoosh H., Baer R., Gu W. (2015). Ferroptosis as a p53-mediated activity during tumour suppression. Nature.

[18] Tarangelo A., Magtanong L., Bieging-Rolett K.T., Li Y., Ye J., Attardi L.D., Dixon S.J. (2018). p53 Suppresses Metabolic Stress-Induced Ferroptosis in Cancer Cells. Cell Rep..

[19] Wang S.J., Li D., Ou Y., Jiang L., Chen Y., Zhao Y., Gu W. (2016). Acetylation Is Crucial for p53-Mediated Ferroptosis and Tumor Suppression. Cell Rep..

[20] Jennis M., Kung C.P., Basu S., Budina-Kolomets A., Leu J.I., Khaku S., Scott J.P., Cai K.Q., Campbell M.R., Porter D.K. (2016). An African-specific polymorphism in the TP53 gene impairs p53 tumor suppressor function in a mouse model. Genes Dev..

[21] Freed-Pastor W.A., Prives C. (2012). Mutant p53: One name, many proteins. Genes Dev..

[22] Clarke S.L., Thompson L.R., Dandekar E., Srinivasan A., Montgomery M.R. (2019). Distinct TP53 Mutation Subtypes Differentially Influence Cellular Iron Metabolism. Nutrients.

[23] Stockwell B.R., Friedmann Angeli J.P., Bayir H., Bush A.I., Conrad M., Dixon S.J., Fulda S., Gascon S., Hatzios S.K., Kagan V.E. (2017). Ferroptosis: A Regulated Cell Death Nexus Linking Metabolism, Redox Biology, and Disease. Cell.

[24] Hentze M.W., Kuhn L.C. (1996). Molecular control of vertebrate iron metabolism: mRNA-based regulatory circuits operated by iron, nitric oxide, and oxidative stress. Proc. Natl. Acad. Sci. USA.

[25] Eisenstein R.S. (2000). Iron regulatory proteins and the molecular control of mammalian iron metabolism. Annu. Rev. Nutr..

[26] Hanson E.S., Foot L.M., Leibold E.A. (1999). Hypoxia post-translationally activates iron-regulatory protein 2. J. Biol. Chem..

[27] Jian Z., Li K., Liu L., Zhang Y., Zhou Z., Li C., Gao T. (2011). Heme oxygenase-1 protects human melanocytes from H2O2-induced oxidative stress via the Nrf2-ARE pathway. J. Investig. Dermatol..

[28] Malhotra D., Portales-Casamar E., Singh A., Srivastava S., Arenillas D., Happel C., Shyr C., Wakabayashi N., Kensler T.W., Wasserman W.W. (2010). Global mapping of binding sites for Nrf2 identifies novel targets in cell survival response through ChIP-Seq profiling and network analysis. Nucleic Acids Res..

[29] Bourseau-Guilmain E., Griveau A., Benoit J.P., Garcion E. (2011). The importance of the stem cell marker prominin-1/CD133 in the uptake of transferrin and in iron metabolism in human colon cancer Caco-2 cells. PLoS ONE.

[30] Cao H., Schroeder B., Chen J., Schott M.B., McNiven M.A. (2016). The Endocytic Fate of the Transferrin Receptor Is Regulated by c-Abl Kinase. J. Biol. Chem..

[31] Okazaki F., Matsunaga N., Okazaki H., Azuma H., Hamamura K., Tsuruta A., Tsurudome Y., Ogino T., Hara Y., Suzuki T. (2016). Circadian Clock in a Mouse Colon Tumor Regulates Intracellular Iron Levels to Promote Tumor Progression. J. Biol. Chem..

[32] Xie Y., Zhu S., Song X., Sun X., Fan Y., Liu J., Zhong M., Yuan H., Zhang L., Billiar T.R. (2017). The Tumor Suppressor p53 Limits Ferroptosis by Blocking DPP4 Activity. Cell Rep..

[33] Lui G.Y., Kovacevic Z., Richardson V., Merlot A.M., Kalinowski D.S., Richardson D.R. (2015). Targeting cancer by binding iron: Dissecting cellular signaling pathways. Oncotarget.

[34] Merlot A.M., Kalinowski D.S., Richardson D.R. (2013). Novel chelators for cancer treatment: Where are we now?. Antioxid. Redox Signal..

[35] Torti S.V., Manz D.H., Paul B.T., Blanchette-Farra N., Torti F.M. (2018). Iron and Cancer. Annu. Rev. Nutr..

[36] Wang Y.Q., Chang S.Y., Wu Q., Gou Y.J., Jia L., Cui Y.M., Yu P., Shi Z.H., Wu W.S., Gao G. (2016). The Protective Role of Mitochondrial Ferritin on Erastin-Induced Ferroptosis. Front. Aging Neurosci..

[37] Alvarez S.W., Sviderskiy V.O., Terzi E.M., Papagiannakopoulos T., Moreira A.L., Adams S., Sabatini D.M., Birsoy K., Possemato R. (2017). NFS1 undergoes positive selection in lung tumours and protects cells from ferroptosis. Nature.

[38] Latunde-Dada G.O. (2017). Ferroptosis: Role of lipid peroxidation, iron and ferritinophagy. Biochim. Biophys. Acta Gen. Subj..

[39] Dixon S.J., Stockwell B.R. (2019). The Hallmarks of Ferroptosis. Annu. Rev. Cancer Biol..

[40] Liu D.S., Duong C.P., Haupt S., Montgomery K.G., House C.M., Azar W.J., Pearson H.B., Fisher O.M., Read M., Guerra G.R. (2017). Inhibiting the system xC(-)/glutathione axis selectively targets cancers with mutant-p53 accumulation. Nat. Commun..

[41] Yoshikawa K., Hamada J., Tada M., Kameyama T., Nakagawa K., Suzuki Y., Ikawa M., Hassan N.M., Kitagawa Y., Moriuchi T. (2010). Mutant p53 R248Q but not R248W enhances in vitro invasiveness of human lung cancer NCI-H1299 cells. Biomed. Res..

[42] Davis M.R., Shawron K.M., Rendina E., Peterson S.K., Lucas E.A., Smith B.J., Clarke S.L. (2011). Hypoxia inducible factor-2 alpha is translationally repressed in response to dietary iron deficiency in Sprague-Dawley rats. J. Nutr..

[43] Schneider C.A., Rasband W.S., Eliceiri K.W. (2012). NIH Image to ImageJ: 25 years of image analysis. Nat. Methods.

[44] Wang W., Di X., D’Agostino R.B., Torti S.V., Torti F.M. (2007). Excess capacity of the iron regulatory protein system. J. Biol. Chem..

[45] Pomerantz M.M., Shrestha Y., Flavin R.J., Regan M.M., Penney K.L., Mucci L.A., Stampfer M.J., Hunter D.J., Chanock S.J., Schafer E.J. (2010). Analysis of the 10q11 cancer risk locus implicates MSMB and NCOA4 in human prostate tumorigenesis. PLoS Genet..

[46] Khritankova I.V., Kukharskiy M.S., Lytkina O.A., Bachurin S.O., Shorning B.Y. (2012). Dimebon activates autophagosome components in human neuroblastoma SH-SY5Y cells. Dokl. Biochem. Biophys..

[47] Yuan H., Li X., Zhang X., Kang R., Tang D. (2016). CISD1 inhibits ferroptosis by protection against mitochondrial lipid peroxidation. Biochem. Biophys. Res. Commun..

[48] Koppula P., Zhang Y., Shi J., Li W., Gan B. (2017). The glutamate/cystine antiporter SLC7A11/xCT enhances cancer cell dependency on glucose by exporting glutamate. J. Biol. Chem..

