# Supplementary material for: Distinct TP53 Mutation Types Exhibit Increased Sensitivity to Ferroptosis Independently of Changes in Iron Regulatory Protein Activity
Source: Int J Mol Sci. 2020 Sep 15;21(18):6751. doi: 10.3390/ijms21186751 (PMC7555626; doi:10.3390/ijms21186751)
Supplement: Supplementary file 1 [file ijms-21-06751-s001.zip › Supplemental data/Figure S1 Raw Gel Shift Images for Manuscript.pptx]

## Slide 1
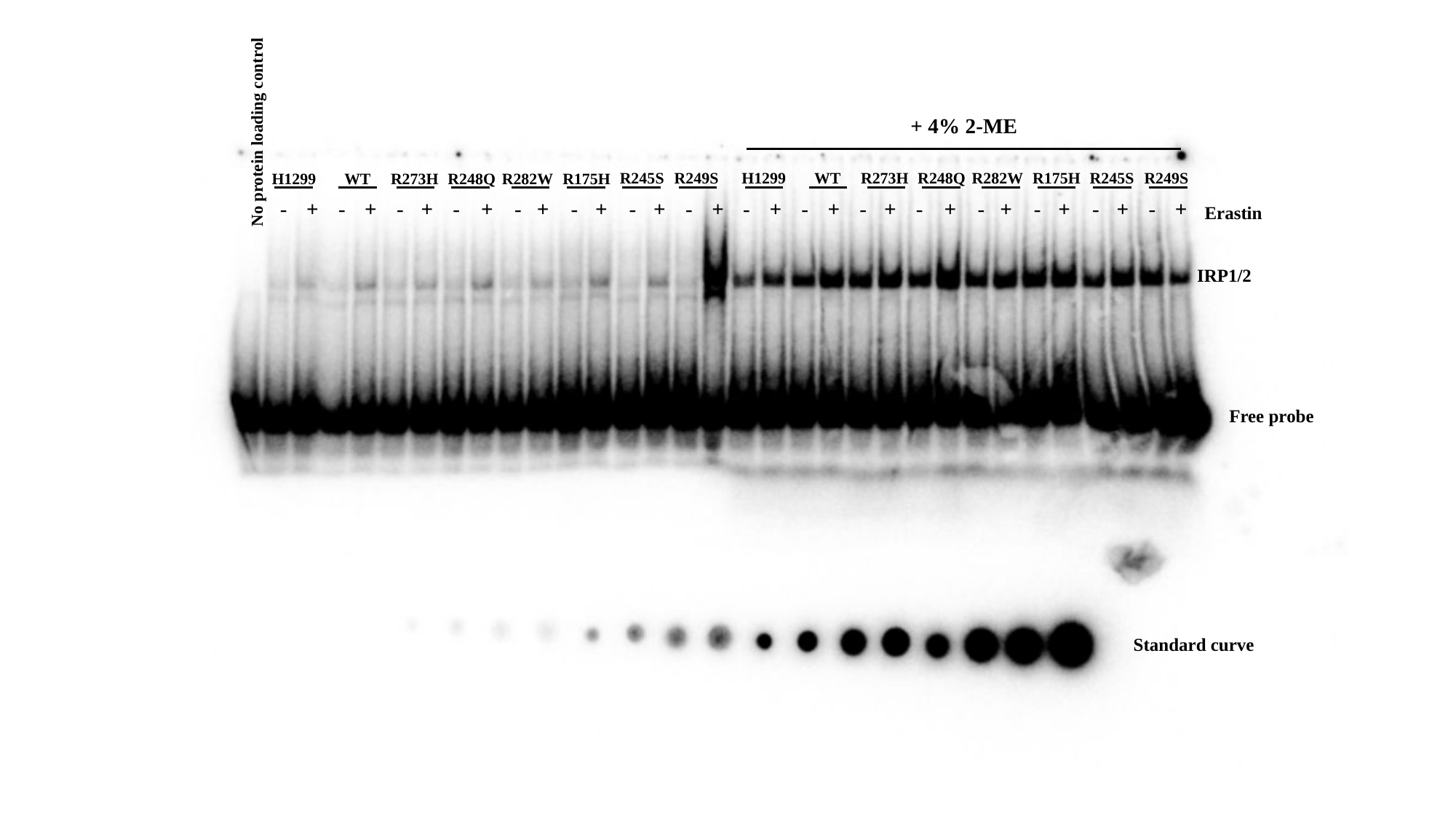

+ 4% 2-ME
No protein loading control
R245S
R249S
R175H
H1299
WT
R273H
R248Q
R282W
R245S
R249S
R175H
H1299
WT
R273H
R248Q
R282W
-
+
-
+
-
+
-
+
-
+
-
+
-
+
-
+
-
+
-
+
-
+
-
+
-
+
-
+
-
+
-
+
Erastin
IRP1/2
Free probe
Standard curve

## Slide 2
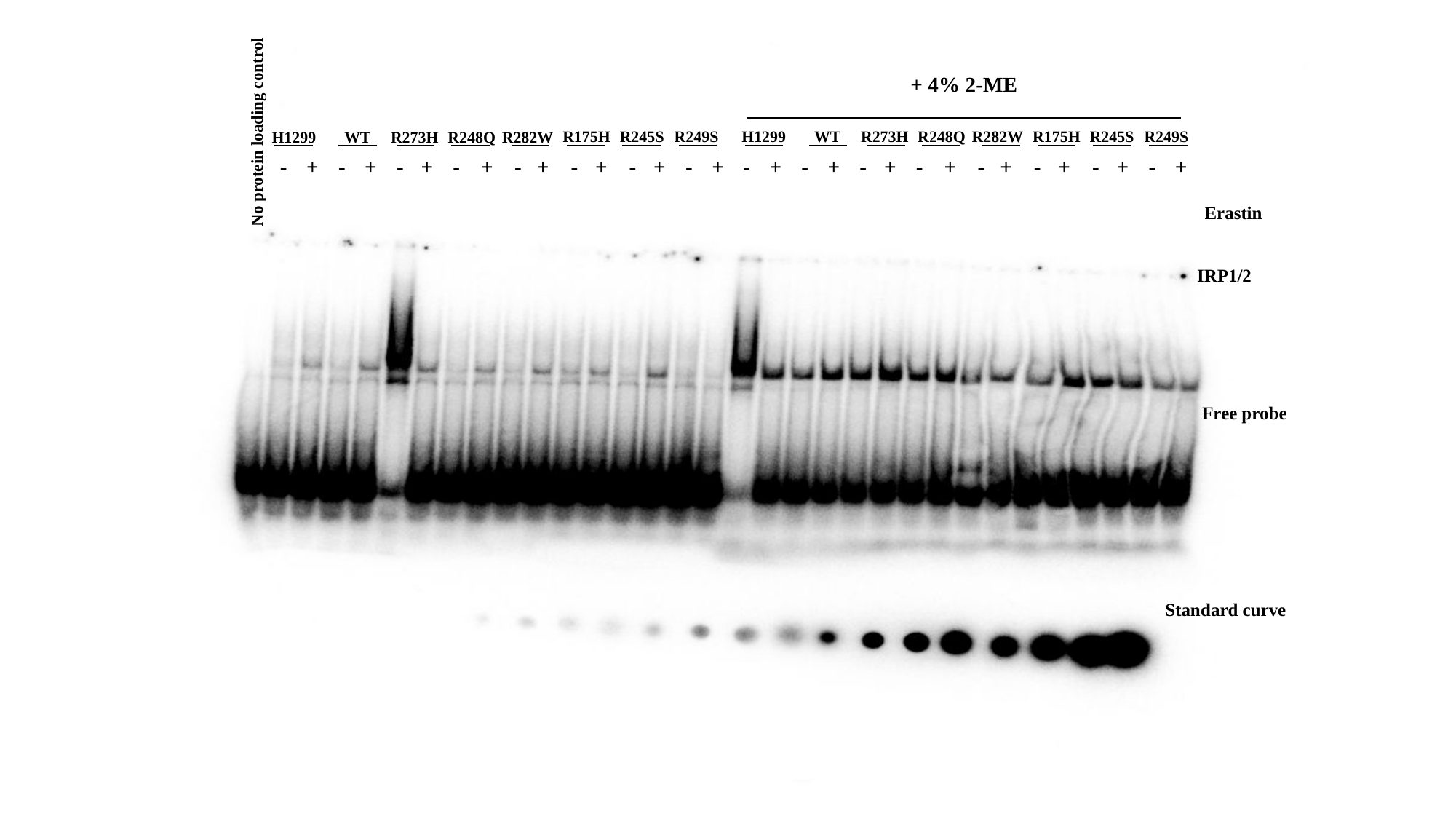

+ 4% 2-ME
No protein loading control
R245S
R249S
R175H
H1299
WT
R273H
R248Q
R282W
R245S
R249S
R175H
H1299
WT
R273H
R248Q
R282W
-
+
-
+
-
+
-
+
-
+
-
+
-
+
-
+
-
+
-
+
-
+
-
+
-
+
-
+
-
+
-
+
Erastin
IRP1/2
Free probe
Standard curve

## Slide 3
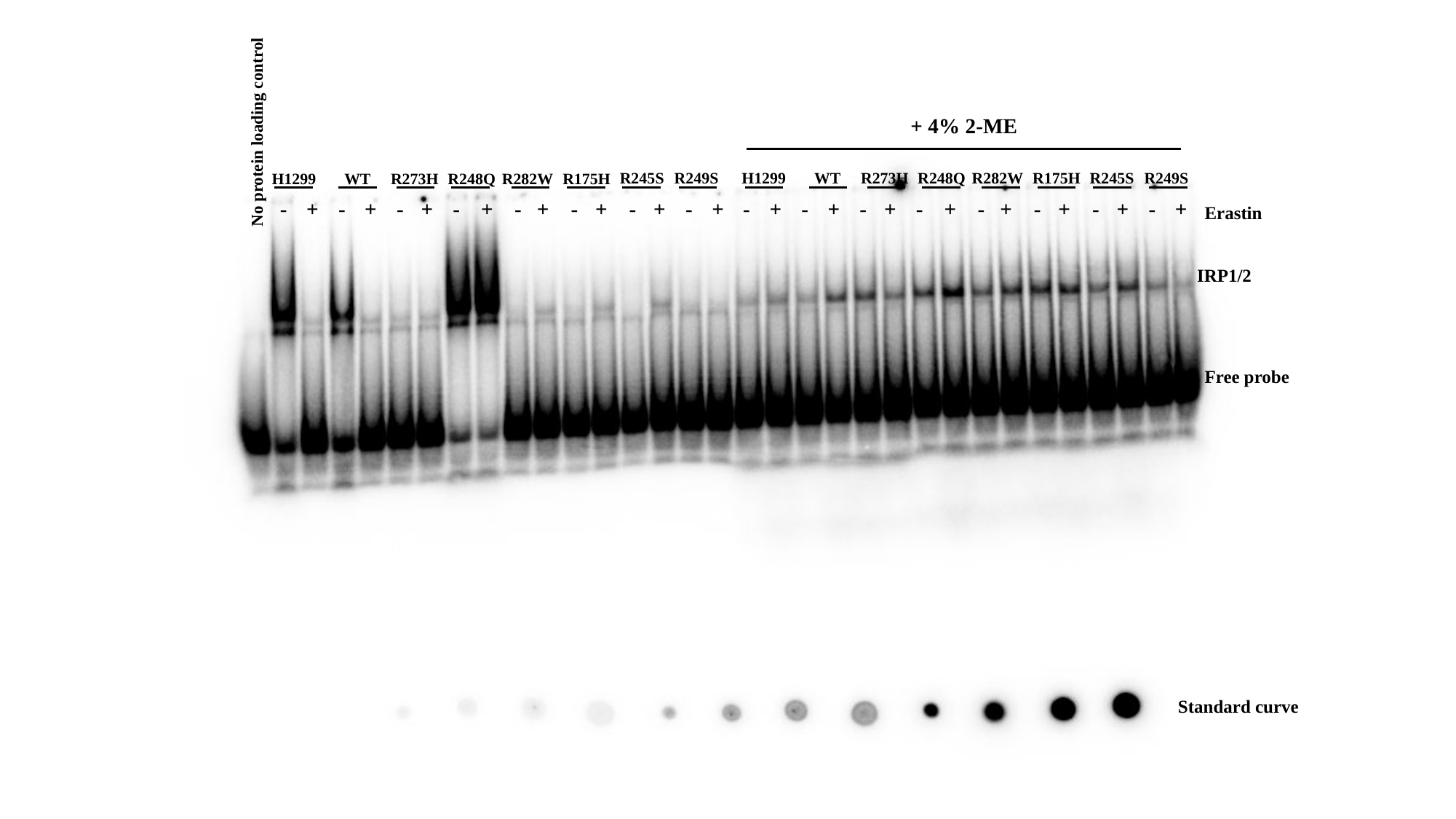

+ 4% 2-ME
No protein loading control
R245S
R249S
R175H
H1299
WT
R273H
R248Q
R282W
R245S
R249S
R175H
H1299
WT
R273H
R248Q
R282W
-
+
-
+
-
+
-
+
-
+
-
+
-
+
-
+
-
+
-
+
-
+
-
+
-
+
-
+
-
+
-
+
Erastin
IRP1/2
Free probe
Standard curve
