# Supplementary material for: Distinct TP53 Mutation Types Exhibit Increased Sensitivity to Ferroptosis Independently of Changes in Iron Regulatory Protein Activity
Source: Int J Mol Sci. 2020 Sep 15;21(18):6751. doi: 10.3390/ijms21186751 (PMC7555626; doi:10.3390/ijms21186751)
Supplement: Supplementary file 1 [file ijms-21-06751-s001.zip › Supplemental data/Figure S2 Raw Western Blot Images for Manuscript.pptx]

## Slide 1
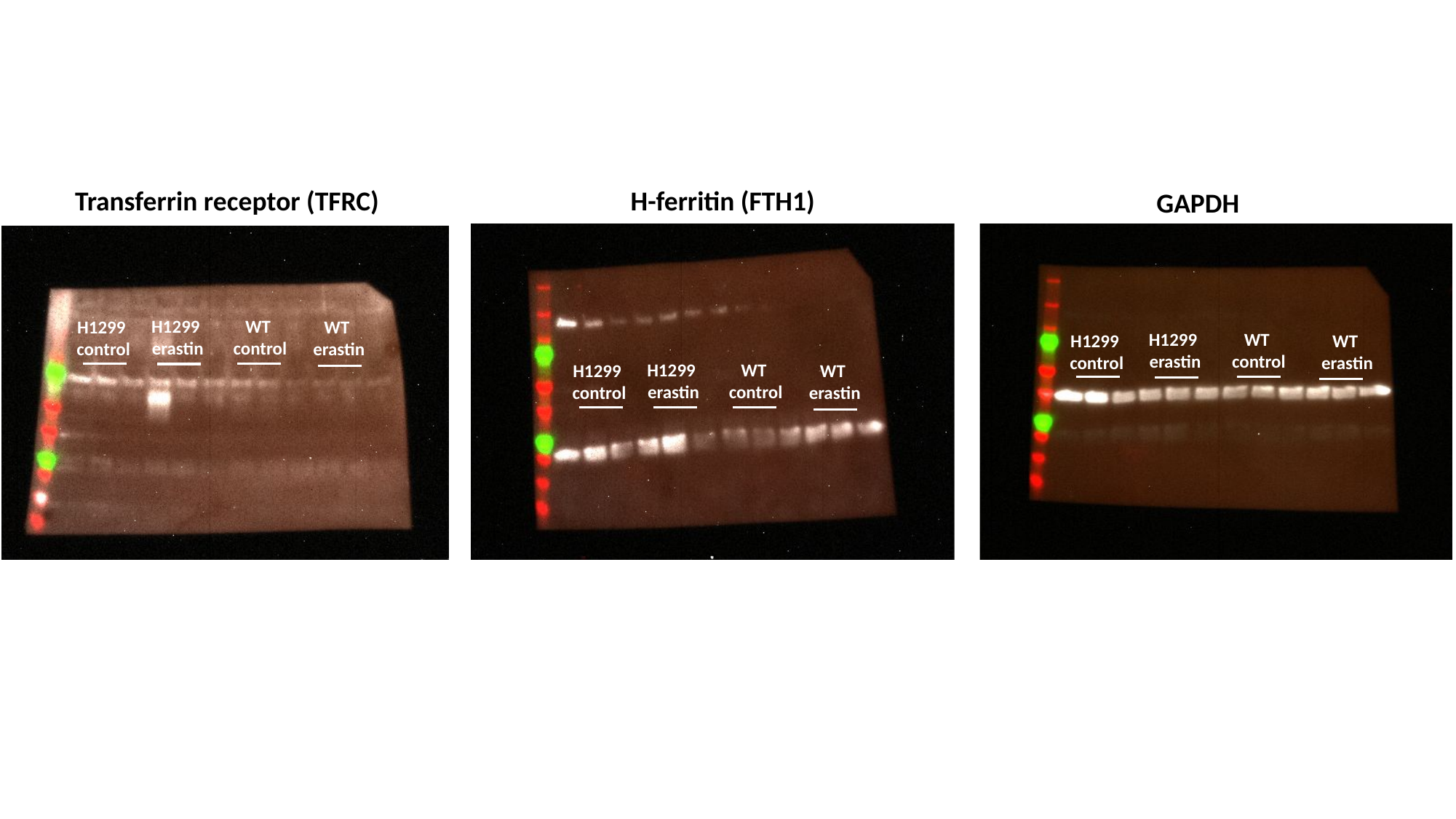

Transferrin receptor (TFRC)
H-ferritin (FTH1)
GAPDH
H1299
erastin
WT
control
H1299
control
WT
erastin
H1299
erastin
WT
control
H1299
control
WT
erastin
H1299
erastin
WT
control
H1299
control
WT
erastin

## Slide 2
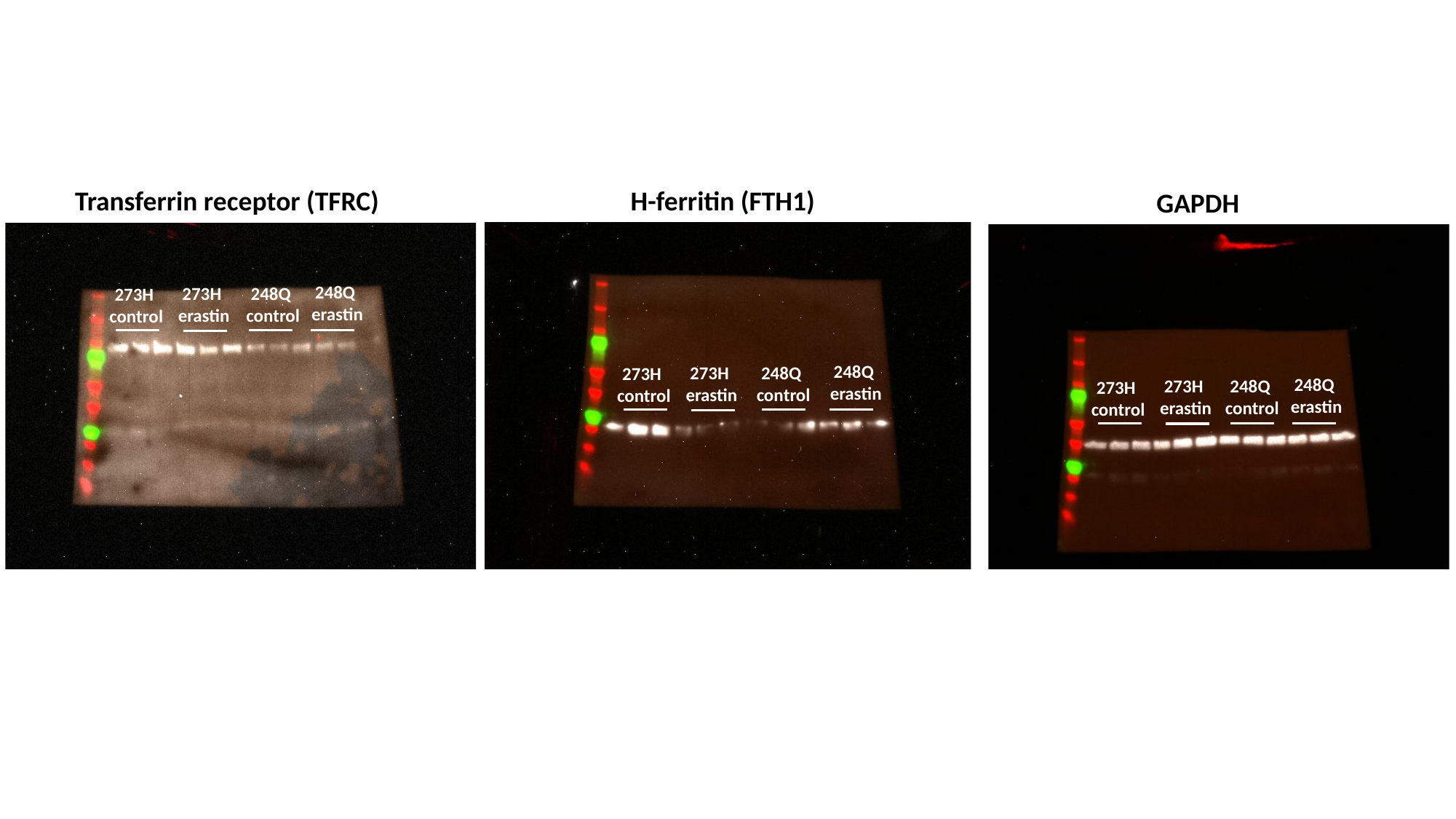

Transferrin receptor (TFRC)
H-ferritin (FTH1)
GAPDH
248Q
erastin
273H
erastin
248Q
control
273H
control
248Q
erastin
273H
erastin
248Q
control
273H
control
248Q
erastin
273H
erastin
248Q
control
273H
control

## Slide 3
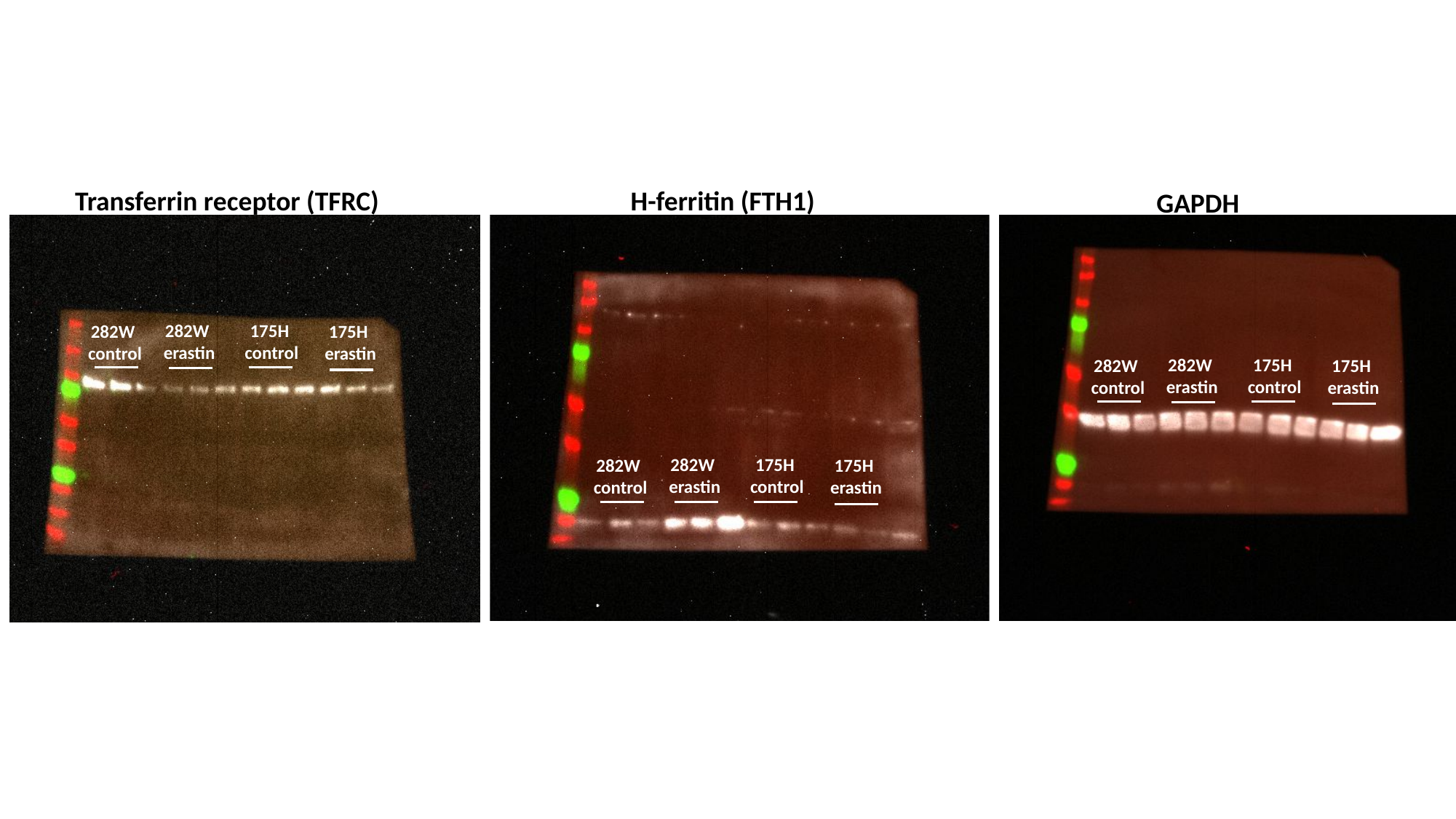

Transferrin receptor (TFRC)
H-ferritin (FTH1)
GAPDH
282W
erastin
175H
control
282W
control
175H
erastin
282W
erastin
175H
control
282W
control
175H
erastin
282W
erastin
175H
control
282W
control
175H
erastin

## Slide 4
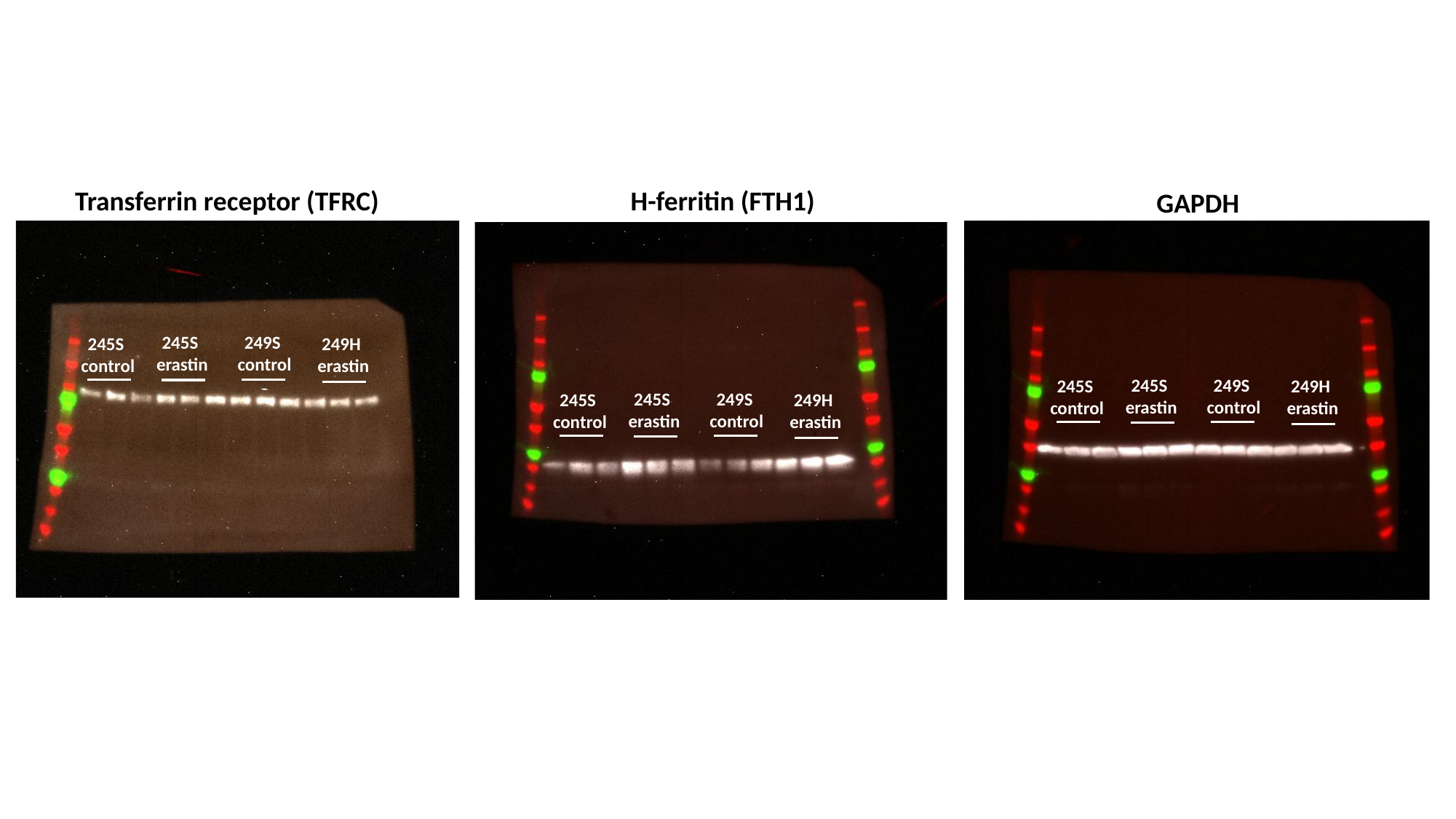

Transferrin receptor (TFRC)
H-ferritin (FTH1)
GAPDH
245S
erastin
249S
control
245S
control
249H
erastin
245S
erastin
249S
control
245S
control
249H
erastin
245S
erastin
249S
control
245S
control
249H
erastin
