# Supplementary material for: Distinct TP53 Mutation Types Exhibit Increased Sensitivity to Ferroptosis Independently of Changes in Iron Regulatory Protein Activity
Source: Int J Mol Sci. 2020 Sep 15;21(18):6751. doi: 10.3390/ijms21186751 (PMC7555626; doi:10.3390/ijms21186751)
Supplement: Supplementary file 1 [file ijms-21-06751-s001.zip › Supplemental data/Supplementary File 11/U9202EC110-1_COA.pdf]

Certificate of Analysis

Project ID: U9202EC110-1

Construct Information:

Gene Name: TP53_(R249S)

Clone ID: I52118 **Gene Length:** 1194 bp

Cloning Vector: pcDNA5-TO **Cloning Strategy:** CloneEZ

QC Items	Specifications	Results	
**Sequencing Alignment**	Sequencing results are consistent with the targeted insert sequence.	Pass	Consistent
**Vector Sequence**	The flanking sequences of the cloning site are correct.	Pass	Correct<br>Shown in the SQD file
**Restriction Digests**	The size of inserted fragment is correct and free of unexpected bands suggesting contamination.	Pass	Correct<br>Shown in attachment 1
**DNA Quality**	Miniprep: 4 μg<br>OD260/280=1.8~2.0<br>Free of contamination	Pass	⩾ 4 μg<br>OD260/280=1.86<br>Pure
**Quality grade**	Research Grade	Pass	Research Grade
**Appearance**	Clear and free of foreign particles.	Pass	Clear<br>Free of foreign particles
**Additional Test**		N/A	

NOTE

Shipping at	Plasmid Storing at	Bacstab Storing at	Glycerol Stock Storing at
Room Temperature	-20℃	4℃	-20℃/-80℃

Morgan

Certified by: **Date: 03/24/2019**

Thank you for your patronage to our Gene services! To maintain this working relationship, we shall be grateful if you can add our webpage URL into your lab website. As a token of appreciation, you will be rewarded by 1,000 EZcoupon™ points. For more information, please contact us by e-mail at web@genscript.com.

For research use only

Attachment 1

Plasmid Construct Map

The gene was cloned in pcDNA5-TO by CloneEZ.

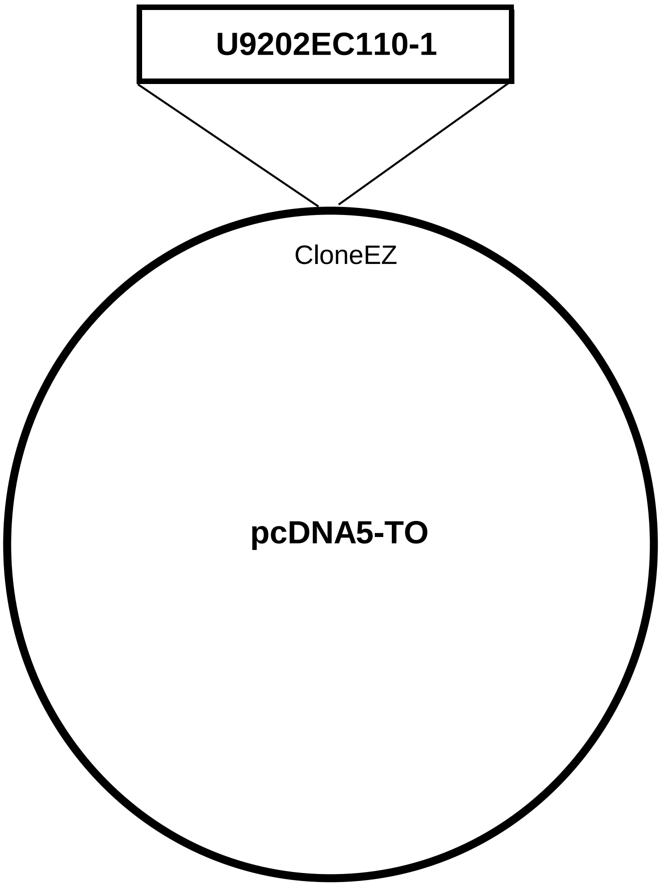

Enzyme Digestion

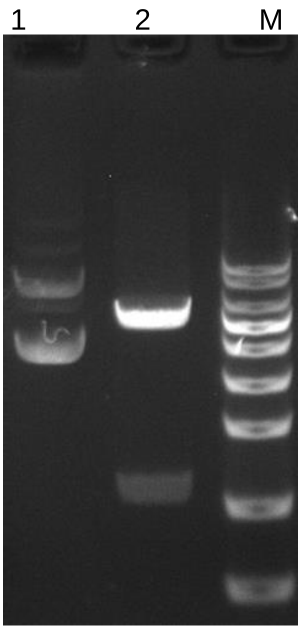

Lane M: KB Ladder
Lane 1:U9202EC110-1 plasmid
Lane 2:U9202EC110-1 plasmid digested by PmeI

Digestion Conditions:
About 300ng plasmid digested
Digestion in water-bath,37℃ for 40 minutes
1% Agarose Gel

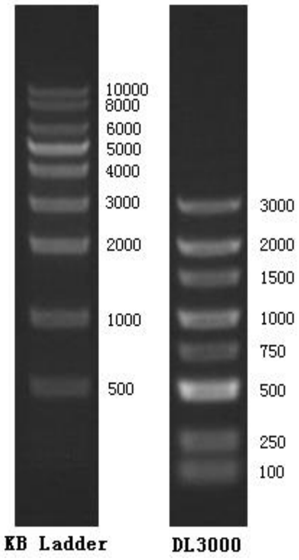

860 Centennial Ave., Piscataway, NJ 08854, USA
Toll-Free: 1-877-436-7274 Tel: 1-732-885-9188 Fax: 1-732-210-0262 Email: order @genscript.com Web: www.genscript.com
