# Supplementary material for: Distinct TP53 Mutation Types Exhibit Increased Sensitivity to Ferroptosis Independently of Changes in Iron Regulatory Protein Activity
Source: Int J Mol Sci. 2020 Sep 15;21(18):6751. doi: 10.3390/ijms21186751 (PMC7555626; doi:10.3390/ijms21186751)

Certificate of Analysis

Project ID: U4129CB060-1

Construct Information:

Gene Symbol: TP53

Clone ID: E92003

Cloning Vector: pcDNA5-TO

Cat No: OHu20059C

Gene Length: 1194 bp

Cloning Strategy: BamHI / XhoI

QC Items	Specifications	Results	
**Sequencing Alignment**	Sequencing results are consistent with the targeted insert sequence.	Pass	Consistent
**Vector Sequence**	The flanking sequences of the cloning site are correct.	Pass	Correct<br>Shown in the SQD file
**Restriction Digests**	The size of inserted fragment is correct and free of unexpected bands suggesting contamination.	Pass	Correct<br>Shown in attachment 1
**DNA Quality**	Miniprep: 10 μg<br>OD260/280=1.8~2.0<br>Free of contamination	Pass	⩾ 10 μg<br>OD260/280=1.83<br>Pure
**Quality grade**	Research Grade	Pass	Research Grade
**Appearance**	Clear and free of foreign particles.	Pass	Clear<br>Free of foreign particles
**Additional Test**		N/A	

NOTE

Shipping at	Plasmid Storing at	Bacstab Storing at	Glycerol Stock Storing at
Room Temperature	-20℃	4℃	-20℃/-80℃

Morgan

Certified by: **Date: 03-14-2017**

Thank you for your patronage to our Gene services! To maintain this working relationship, we shall be grateful if you can add our webpage URL into your lab website. As a token of appreciation, you will be rewarded by 1,000 EZcoupon™ points. For more information, please contact us by e-mail at web@genscript.com.

Attachment 1

Plasmid Construct Map

The gene was cloned in pcDNA5-TO by BamHI / XhoI.

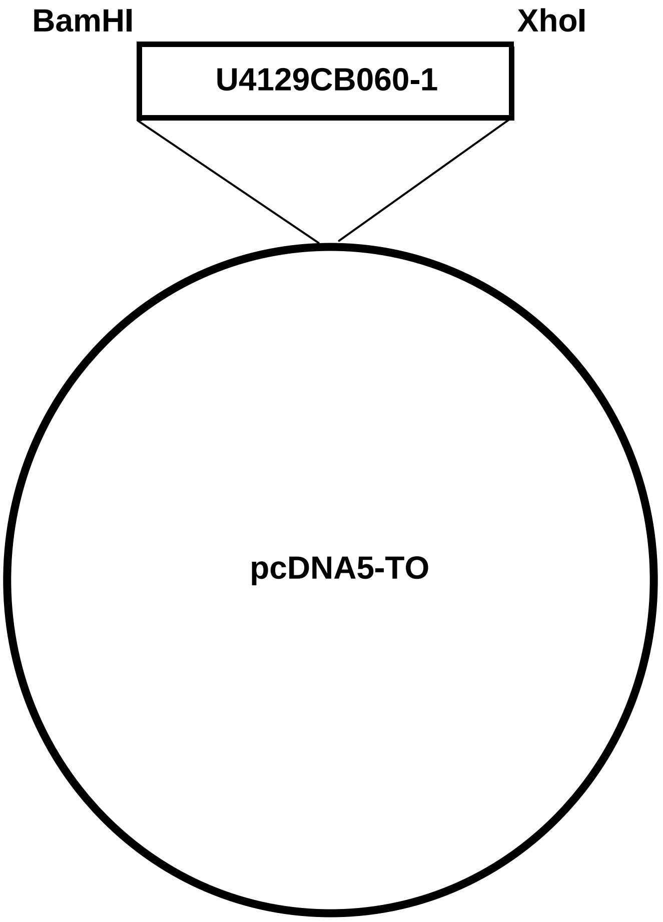

Enzyme Digestion

1 2 M

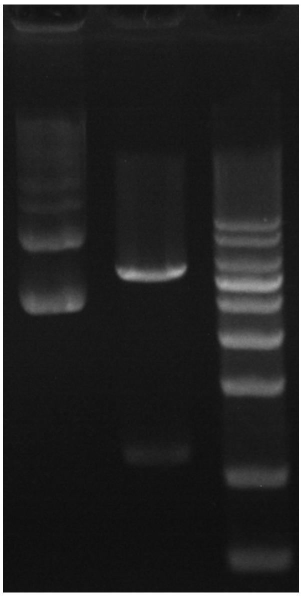

Lane M: KB Ladder
Lane 1:U4129CB060-1 plasmid
Lane 2:U4129CB060-1 plasmid digested
by XhoI and BamHI

Digestion Conditions:
About 300ng plasmid digested
Digestion in water-bath,37℃ for 40 minutes
1% Agarose Gel

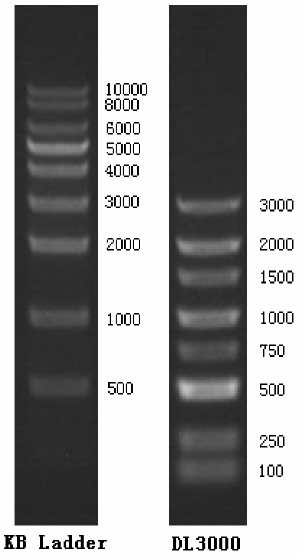

Supplement: Supplementary file 1 [file ijms-21-06751-s001.zip › Supplemental data/Supplementary File 1/U4129CB060-1_COA.pdf]
