# Supplementary material for: Distinct TP53 Mutation Types Exhibit Increased Sensitivity to Ferroptosis Independently of Changes in Iron Regulatory Protein Activity
Source: Int J Mol Sci. 2020 Sep 15;21(18):6751. doi: 10.3390/ijms21186751 (PMC7555626; doi:10.3390/ijms21186751)
Supplement: Supplementary file 1 [file ijms-21-06751-s001.zip › Supplemental data/Supplementary File 6/U4129CB060-6_COA.pdf]

Certificate of Analysis

Project ID: U4129CB060-6

Lot No.: U4129CB060S-6/G121358

Plasmid Name: TP53_(H193Y)_pcDNA5/TO

Quantity: 100 μg(0.1 mg each,1 vial) **Concentration:** 1 mg/mL

Final Volume: 100 μl **Final Buffer:** TE

QC Items	Specifications	Results	
**Appearance**	Colorless, clear, free of precipitate or foreign particles	Pass	Colorless, clear, free of precipitate or foreign particles
**Identity**	Co-migrates with reference DNA and/or size confirmed versus marker	Pass	Confirmed
**Restriction Digests**	Matches expected test	Pass	Matched<br>Shown in attachment 1
**OD260/OD280**	1.8-2.0	Pass	1.89
**Residual RNA**	Not visible on agarose gel	Pass	Invisible
**Genomic DNA**	Not visible on agarose gel	Pass	Invisible
**Endotoxin**	☒ ≤ 0.01 EU/μg<br>☐ No QC testing requested	Pass	≤ 0.01 EU/μg
**Bio-Burden Assay**	☒ Bio-Burden Assay<br>☐ No QC testing requested	Pass	No viable organism
**Additional Test**		N/A	

NOTE

Shipping at	Plasmid Storing at	Bacstab Storing at	Glycerol Stock Storing at
Room Temperature	-20℃	4℃	-20℃/-80℃

Certified Morgan **Date: 2017/3/14**

Thank you for your patronage to our Gene services! To maintain this working relationship, we shall be grateful if you can add our webpage URL into your lab website. As a token of appreciation, you will be rewarded by 1,000 EZcoupon™ points. For more information, please contact us by e-mail at web@genscript.com.

For research use only

Attachment 1

Enzyme Digestion

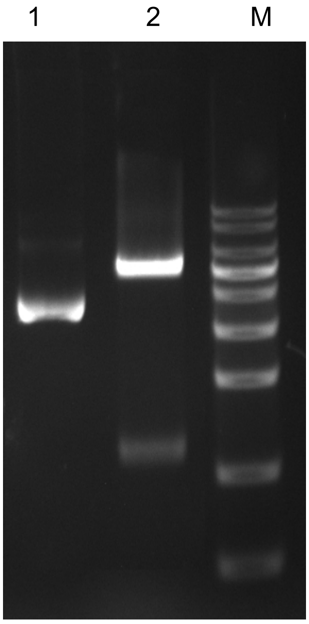

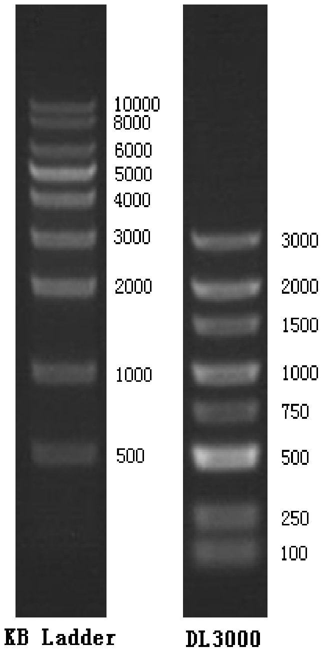

Lane M: KB Ladder
Lane 1: U4129CB060-6 plasmid
Lane 2: U4129CB060-6 plasmid digested
by BamHI and XhoI

Digestion Conditions:
About 300ng plasmid digested
Digestion in water-bath,37℃ for 40 minutes
1% Agarose Gel
