# Supplementary material for: Distinct TP53 Mutation Types Exhibit Increased Sensitivity to Ferroptosis Independently of Changes in Iron Regulatory Protein Activity
Source: Int J Mol Sci. 2020 Sep 15;21(18):6751. doi: 10.3390/ijms21186751 (PMC7555626; doi:10.3390/ijms21186751)
Supplement: Supplementary file 1 [file ijms-21-06751-s001.zip › Supplemental data/Supplementary File 7/U4129CB060-7_COA.pdf]

Certificate of Analysis

Project ID: U4129CB060-7

Construct Information:

Gene Name: TP53 (R248Q)

Clone ID: E92009

Gene Length: 1197 bp

Cloning Vector: pcDNA5/TO

Cloning Strategy: BamHI / XhoI

QC Items	Specifications	Results	
**Sequencing Alignment**	Sequencing results are consistent with the targeted insert sequence.	Pass	Consistent
**Vector Sequence**	The flanking sequences of the cloning site are correct.	Pass	Correct<br>Shown in the SQD file
**Restriction Digests**	The size of inserted fragment is correct and free of unexpected bands suggesting contamination.	Pass	Correct<br>Shown in attachment 1
**DNA Quality**	Miniprep: 4 μg<br>OD260/280=1.8~2.0<br>Free of contamination	Pass	⩾ 4 μg<br>OD260/280=1.88<br>Pure
**Quality grade**	Research Grade	Pass	Research Grade
**Appearance**	Clear and free of foreign particles.	Pass	Clear<br>Free of foreign particles
**Additional Test**		N/A	

NOTE

Shipping at	Plasmid Storing at	Bacstab Storing at	Glycerol Stock Storing at
Room Temperature	-20℃	4℃	-20℃/-80℃

Certified by: Morgan

Date: 03-14-2017

Thank you for your patronage to our Gene services! To maintain this working relationship, we shall be grateful if you can add our webpage URL into your lab website. As a token of appreciation, you will be rewarded by 1,000 EZcoupon™ points. For more information, please contact us by e-mail at web@genscript.com.

860 Centennial Ave., Piscataway, NJ 08854, USA
Toll-Free: 1-877-436-7274 Tel: 1-732-885-9188 Fax: 1-732-210-0262 Email: order@genscript.com Web: www.genscript.com

Attachment 1

Plasmid Construct Map

The gene was cloned in pcDNA5/TO by BamHI / XhoI.

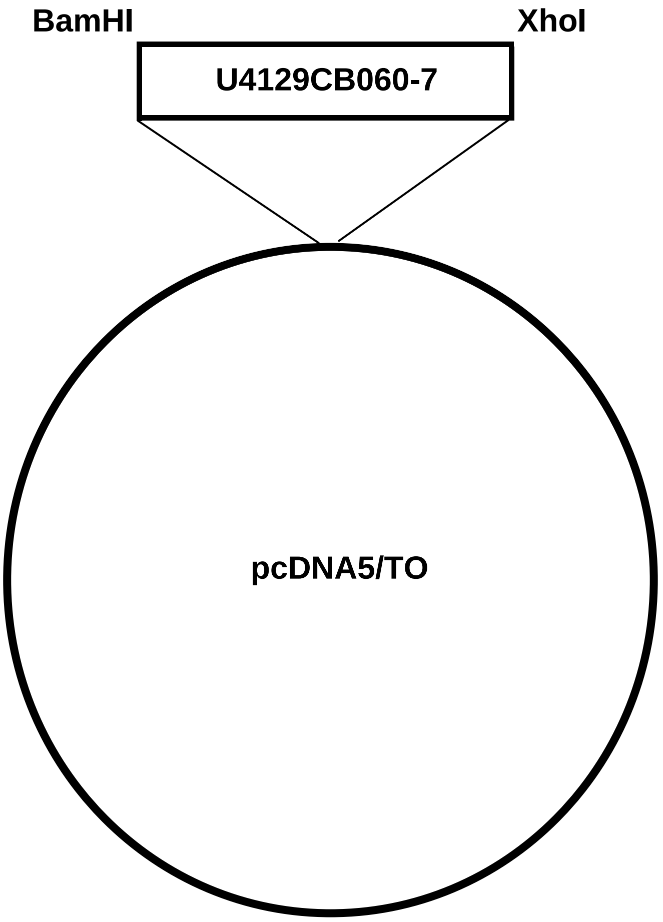

Enzyme Digestion

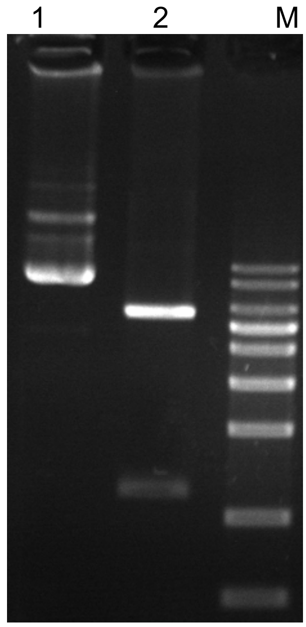

Lane M: KB Ladder
Lane 1:U4129CB060-7 plasmid
Lane 2:U4129CB060-7 plasmid digested by BamHI and XhoI

Digestion Conditions:
About 300ng plasmid digested
Digestion in water-bath,37℃ for 40 minutes
1% Agarose Gel

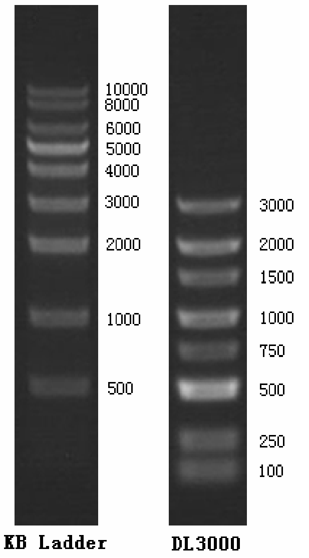

For research use only
