# Supplementary material for: Distinct TP53 Mutation Types Exhibit Increased Sensitivity to Ferroptosis Independently of Changes in Iron Regulatory Protein Activity
Source: Int J Mol Sci. 2020 Sep 15;21(18):6751. doi: 10.3390/ijms21186751 (PMC7555626; doi:10.3390/ijms21186751)
Supplement: Supplementary file 1 [file ijms-21-06751-s001.zip › Supplemental data/Supplementary File 8/U4129CB060-8_COA.pdf]

# Certificate of Analysis

**Project ID:** U4129CB060-8

**Lot No.:** U4129CB060S-8/G121359

**Plasmid Name:** TP53_(R248Q)_pcDNA5/TO

**Quantity:** 100 µg(0.1 mg each,1 vial) **Concentration:** 1 mg/mL

**Final Volume:** 100 µl **Final Buffer:** TE

| QC Items | Specifications | Results | |
|---|---|---|---|
| **Appearance** | Colorless, clear, free of precipitate or foreign particles | Pass | Colorless, clear, free of precipitate or foreign particles |
| **Identity** | Co-migrates with reference DNA and/or size confirmed versus marker | Pass | Confirmed |
| **Restriction Digests** | Matches expected test | Pass | Matched<br>Shown in attachment 1 |
| **OD260/OD280** | 1.8-2.0 | Pass | 1.88 |
| **Residual RNA** | Not visible on agarose gel | Pass | Invisible |
| **Genomic DNA** | Not visible on agarose gel | Pass | Invisible |
| **Endotoxin** | ☒ ≤ 0.01 EU/µg<br>☐ No QC testing requested | Pass | ≤ 0.01 EU/µg |
| **Bio-Burden Assay** | ☒ Bio-Burden Assay<br>☐ No QC testing requested | Pass | No viable organism |
| **Additional Test** | | N/A | |

## NOTE

| Shipping at | Plasmid Storing at | Bacstab Storing at | Glycerol Stock Storing at |
|---|---|---|---|
| Room Temperature | -20℃ | 4℃ | -20℃/-80℃ |

**Certified** Morgan **Date: 2017/3/14**

Thank you for your patronage to our Gene services! To maintain this working relationship, we shall be grateful if you can add our webpage URL into your lab website. As a token of appreciation, you will be rewarded by 1,000 EZcoupon™ points. For more information, please contact us by e-mail at web@genscript.com.

**Attachment 1**

# Enzyme Digestion

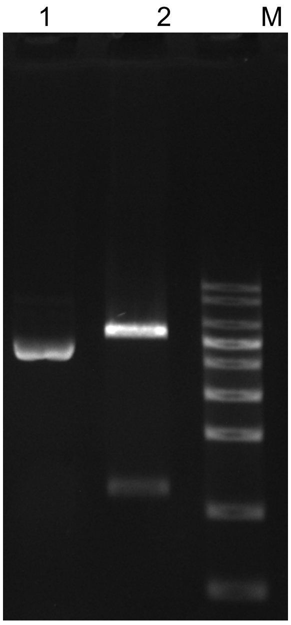


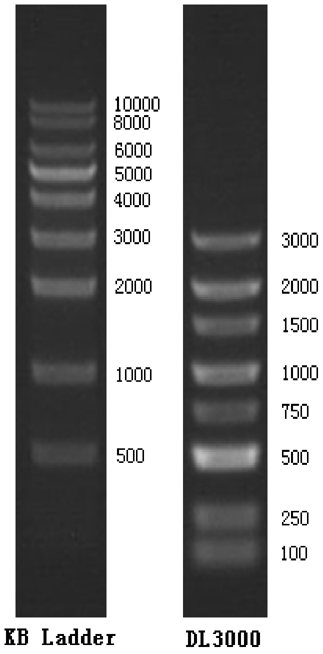


Lane M: KB Ladder
Lane 1: U4129CB060-8 plasmid
Lane 2: U4129CB060-8 plasmid digested by BamHI and XhoI

**Digestion Conditions:**

About 300ng plasmid digested
Digestion in water-bath,37℃ for 40 minutes
1% Agarose Gel
