# Supplementary figures and images for: Distinct TP53 Mutation Types Exhibit Increased Sensitivity to Ferroptosis Independently of Changes in Iron Regulatory Protein Activity
Source: Int J Mol Sci. 2020 Sep 15;21(18):6751. doi: 10.3390/ijms21186751 (PMC7555626; doi:10.3390/ijms21186751)

## Slide 1
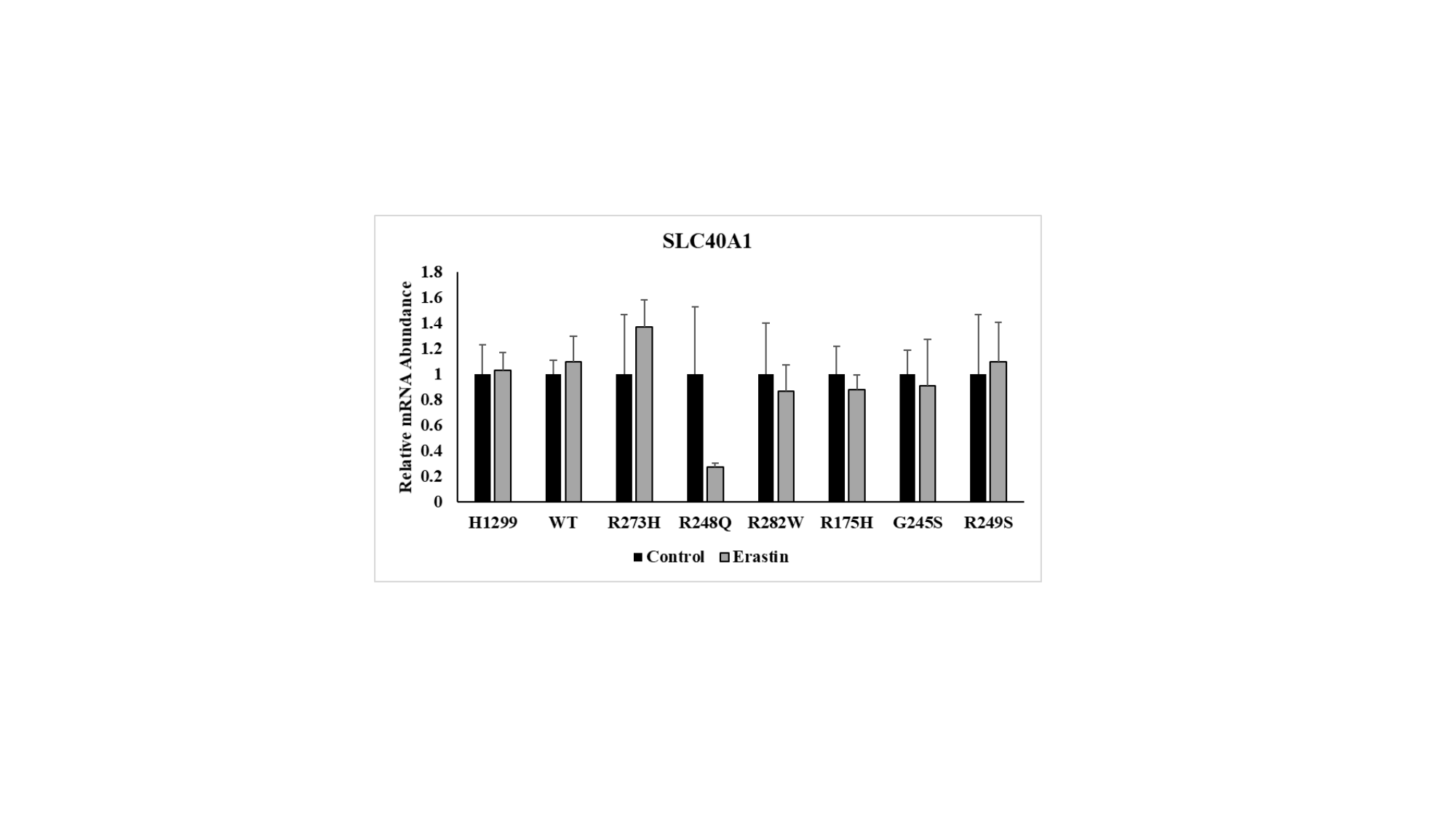

Supplement: Supplementary file 1 [file ijms-21-06751-s001.zip › Supplemental data/Figure S3 Ferroportin (SLC40A1) mRNA expression.pptx]

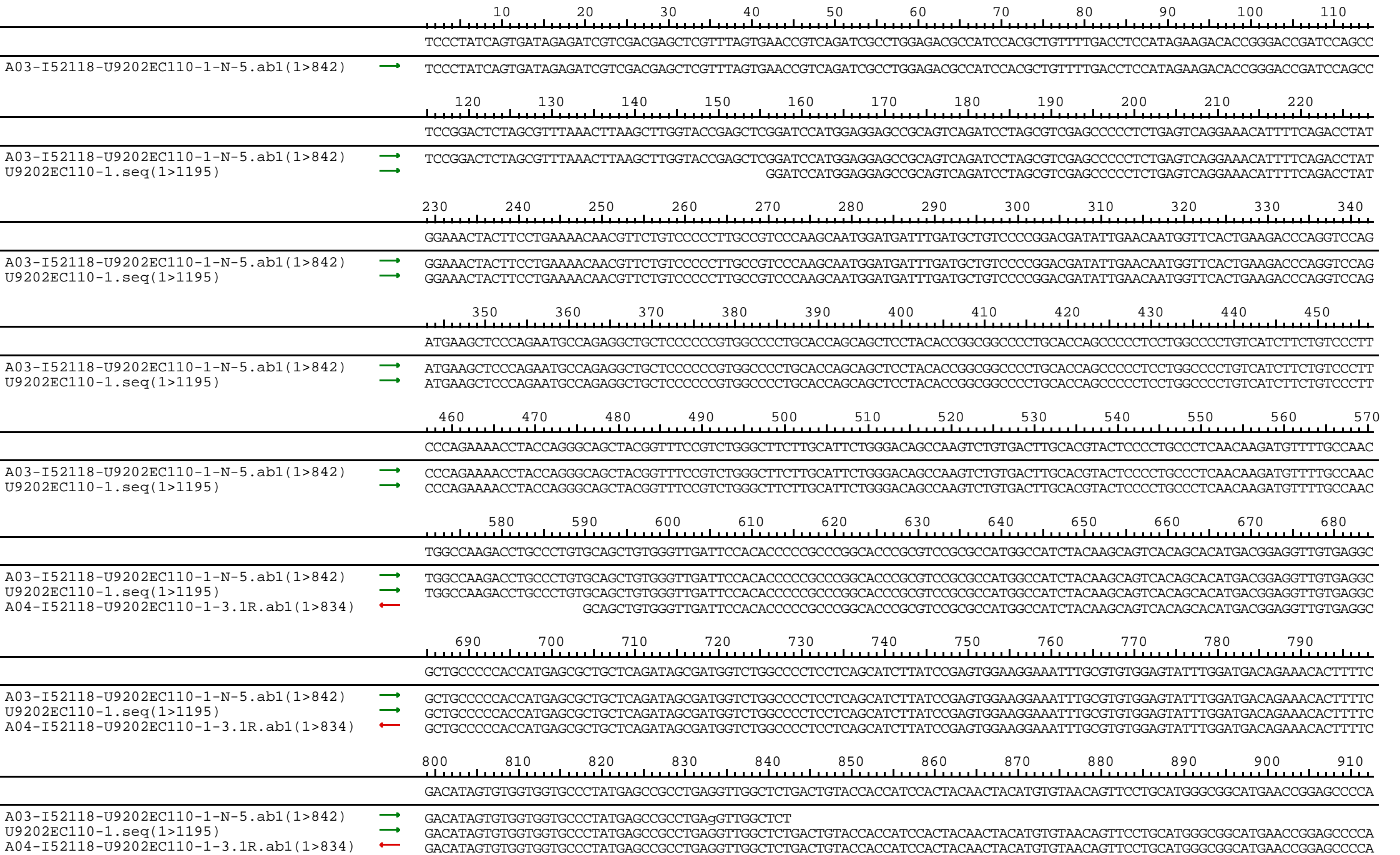

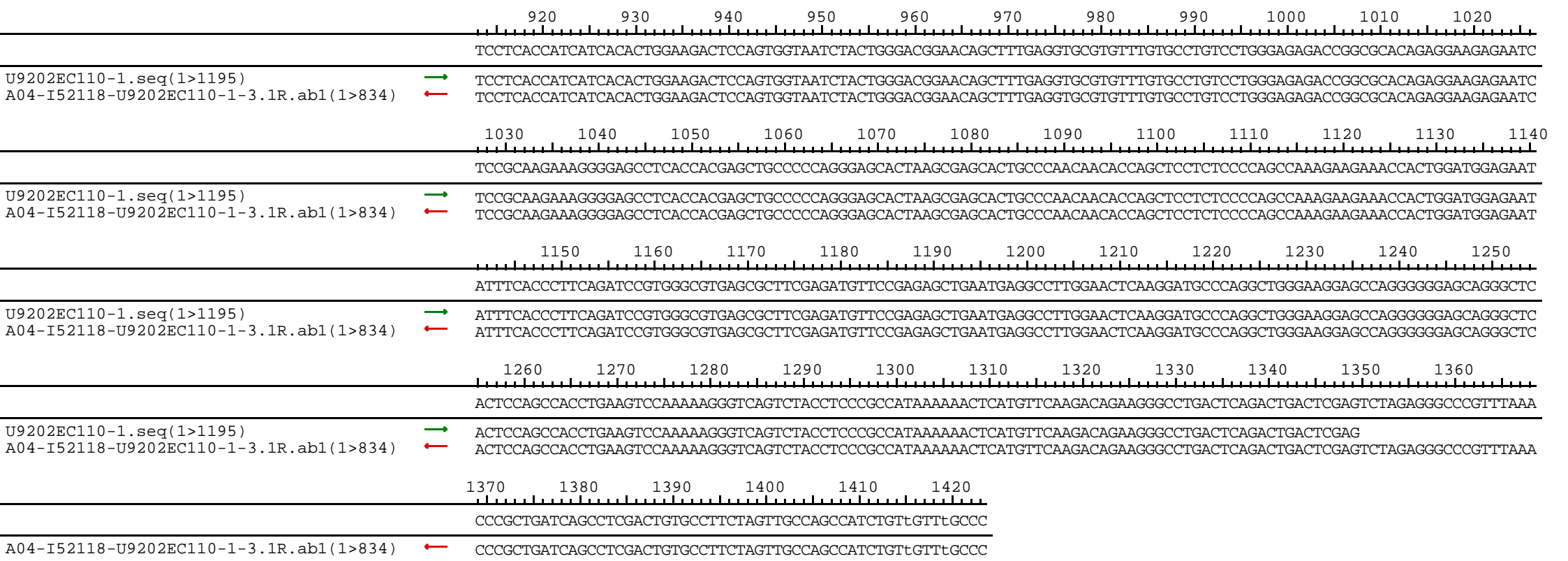

Supplement: Supplementary file 1 [file ijms-21-06751-s001.zip › Supplemental data/Supplementary File 11/U9202EC110-1-sqd.pdf]

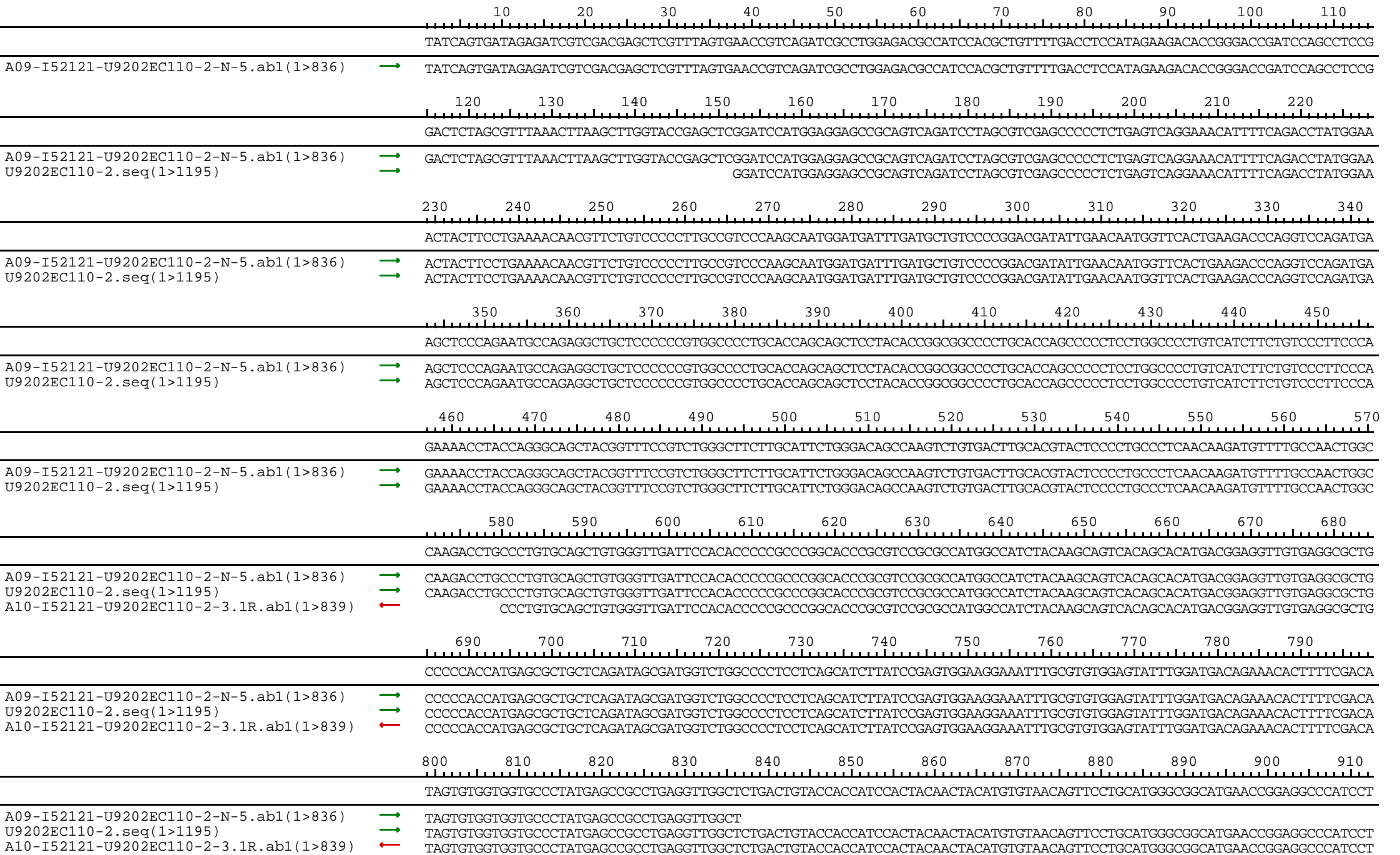

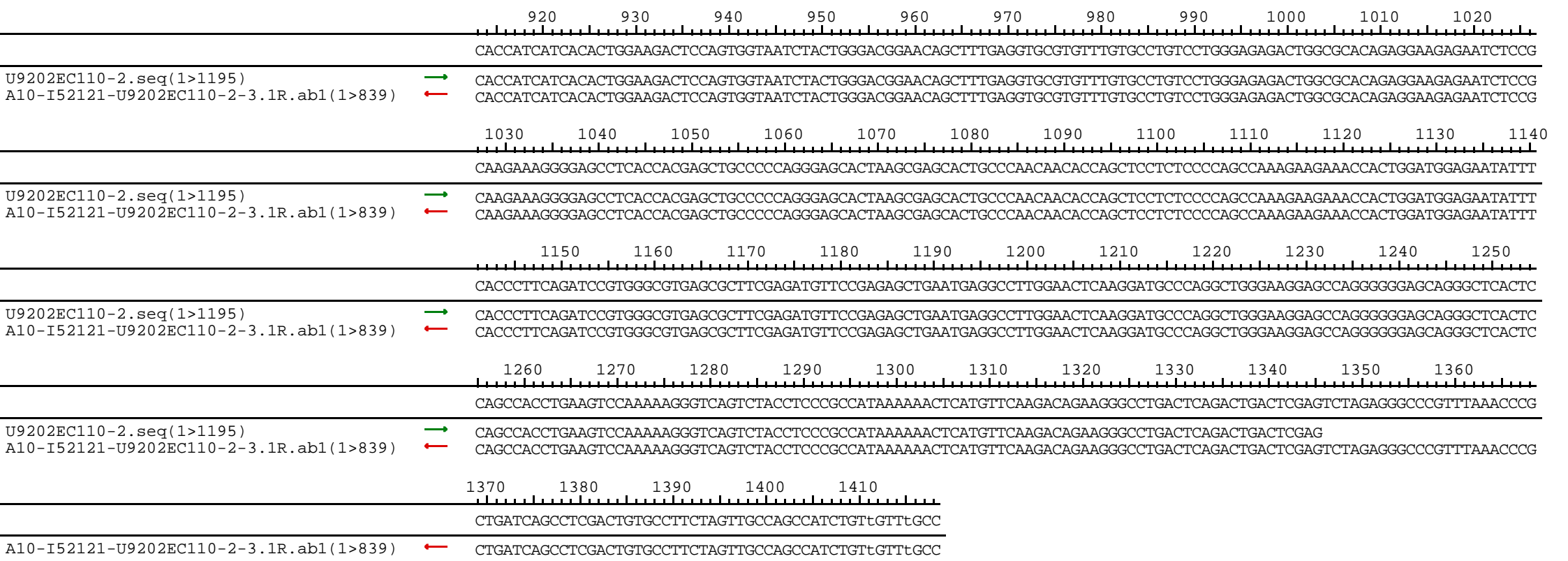

Supplement: Supplementary file 1 [file ijms-21-06751-s001.zip › Supplemental data/Supplementary File 12/U9202EC110-2-sqd.pdf]

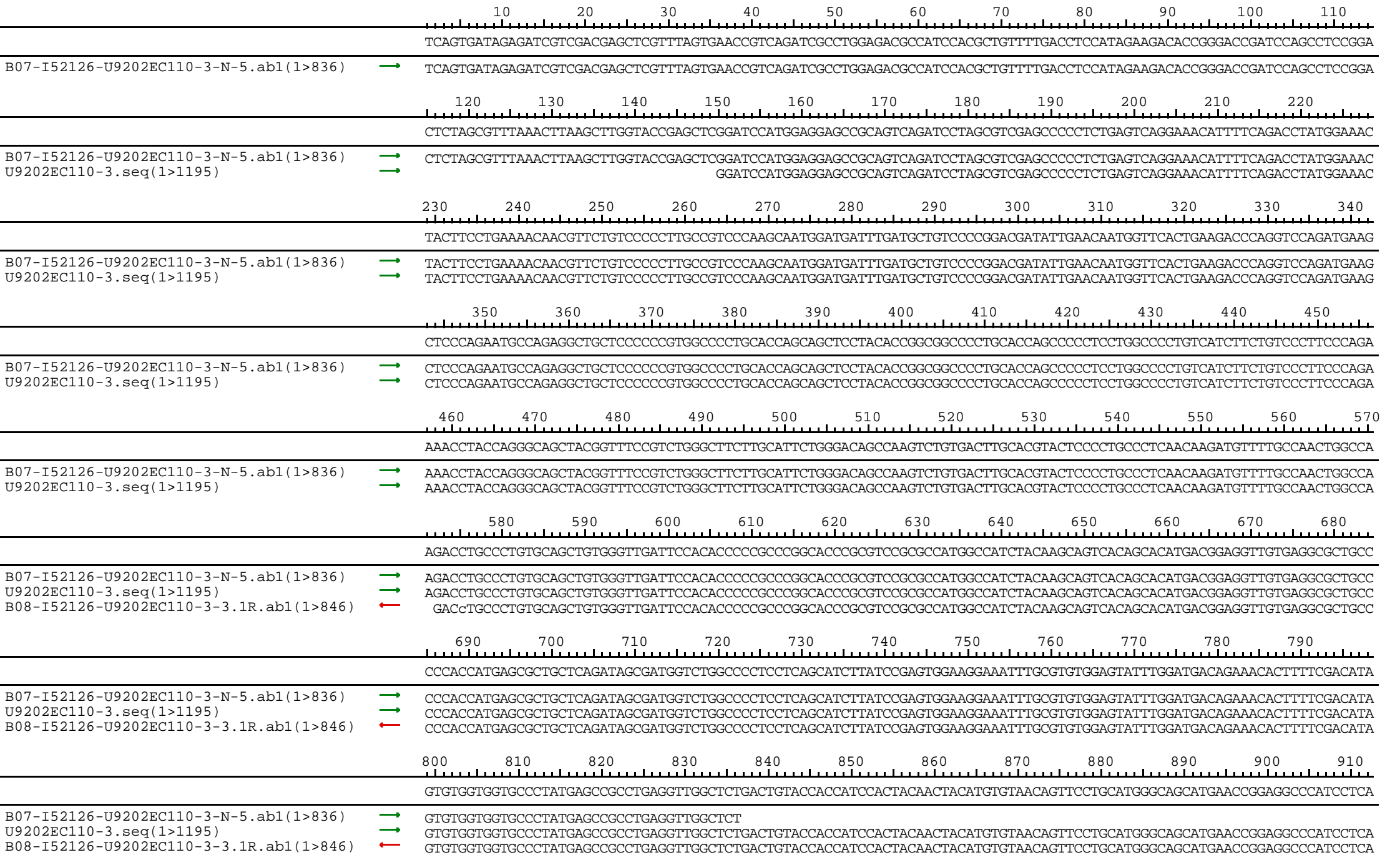

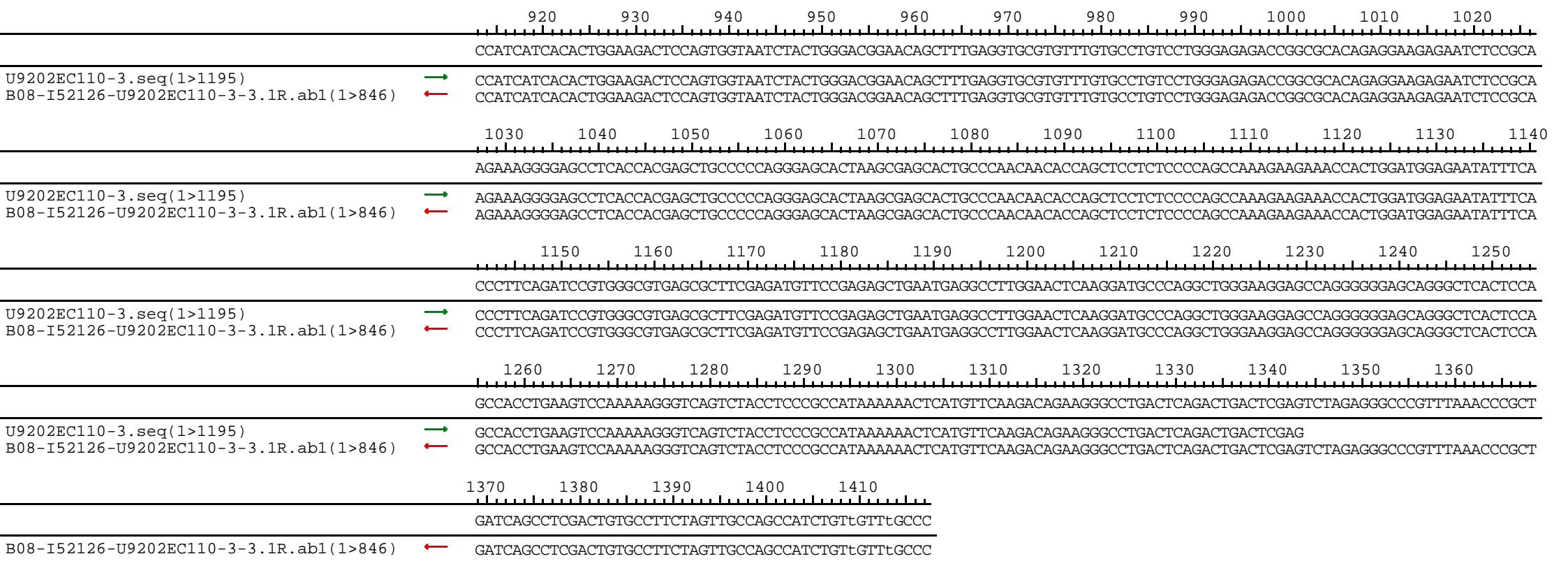

Supplement: Supplementary file 1 [file ijms-21-06751-s001.zip › Supplemental data/Supplementary File 13/U9202EC110-3-sqd.pdf]

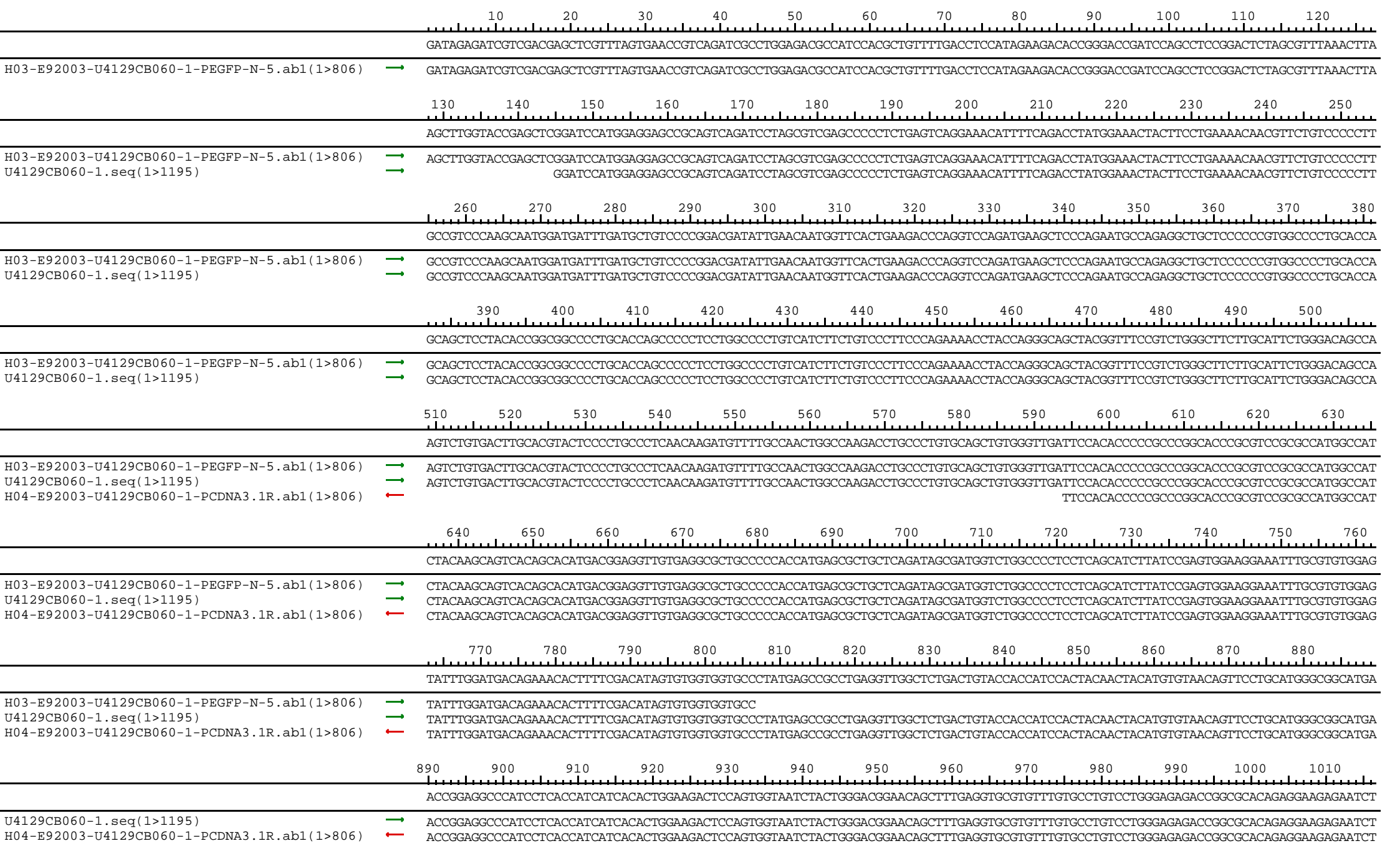

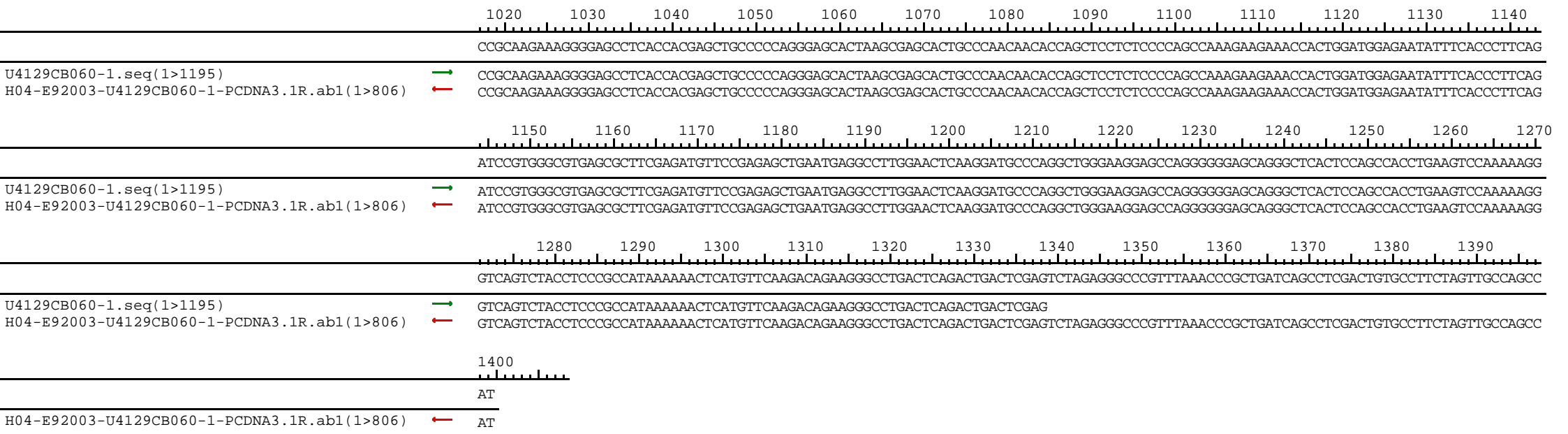

Supplement: Supplementary file 1 [file ijms-21-06751-s001.zip › Supplemental data/Supplementary File 1/U4129CB060-1-sqd.pdf]

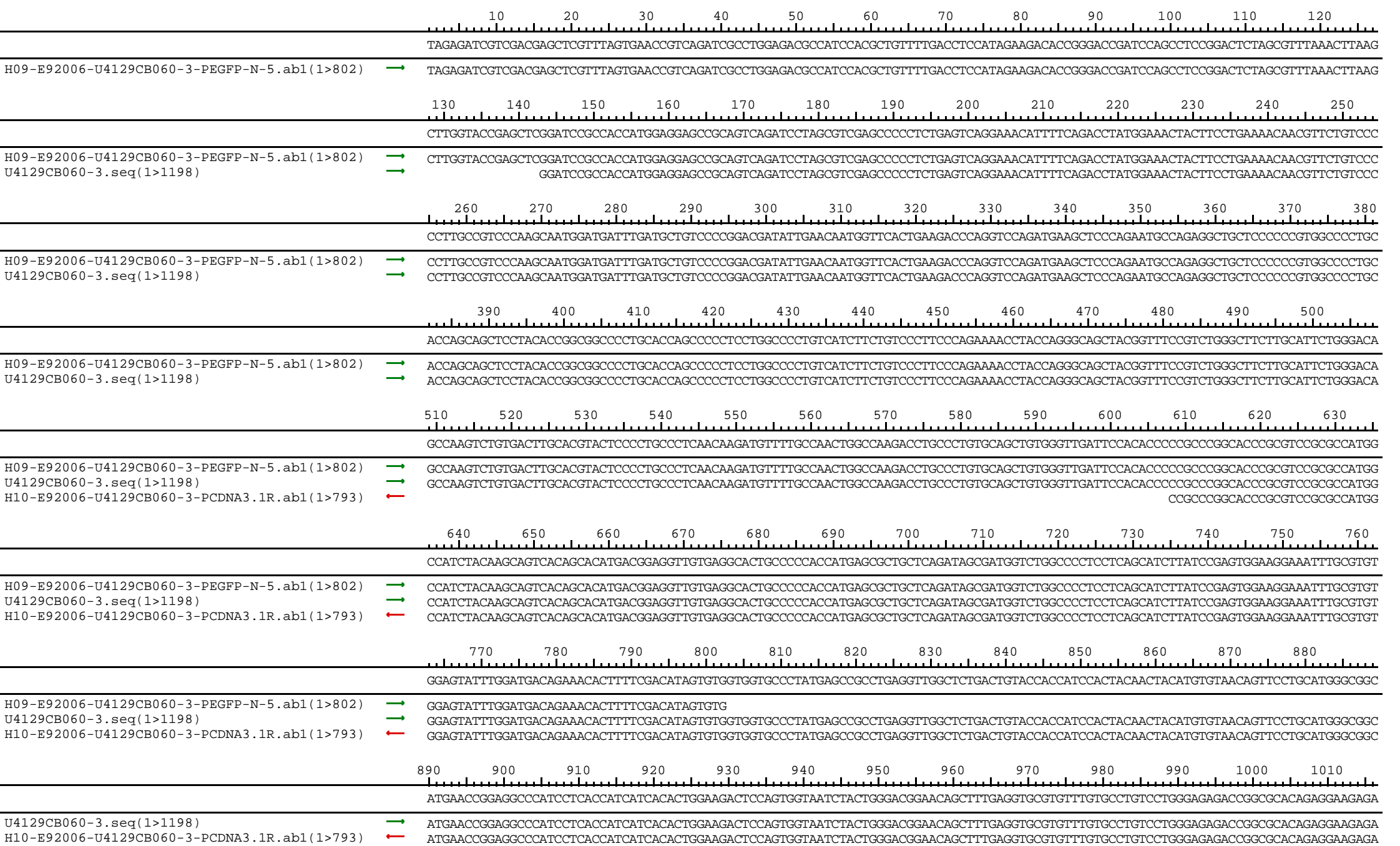

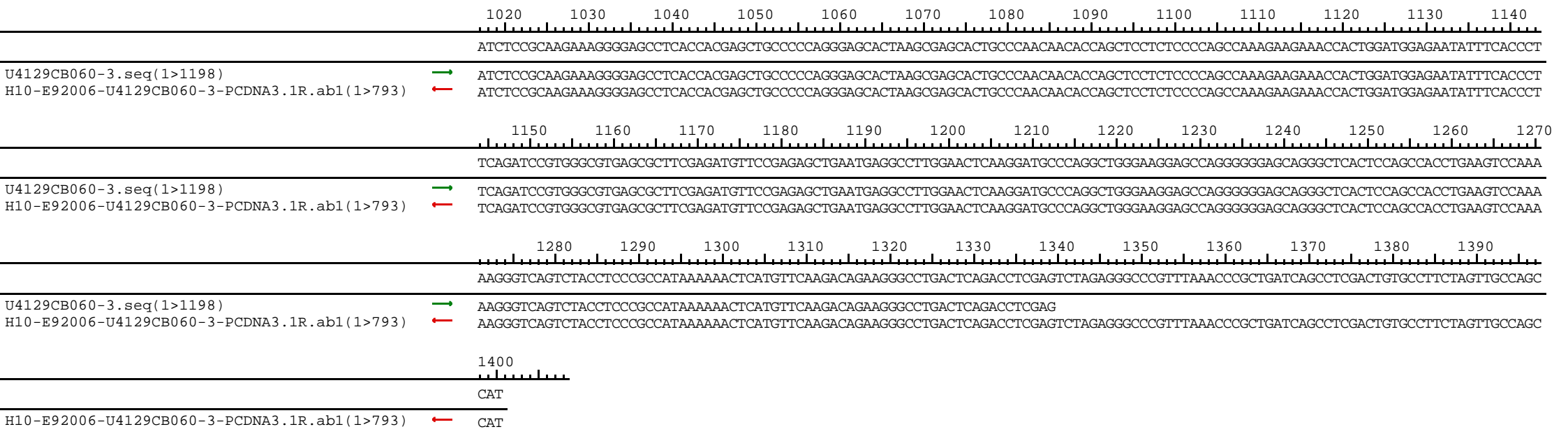

Supplement: Supplementary file 1 [file ijms-21-06751-s001.zip › Supplemental data/Supplementary File 3/U4129CB060-3-sqd.pdf]

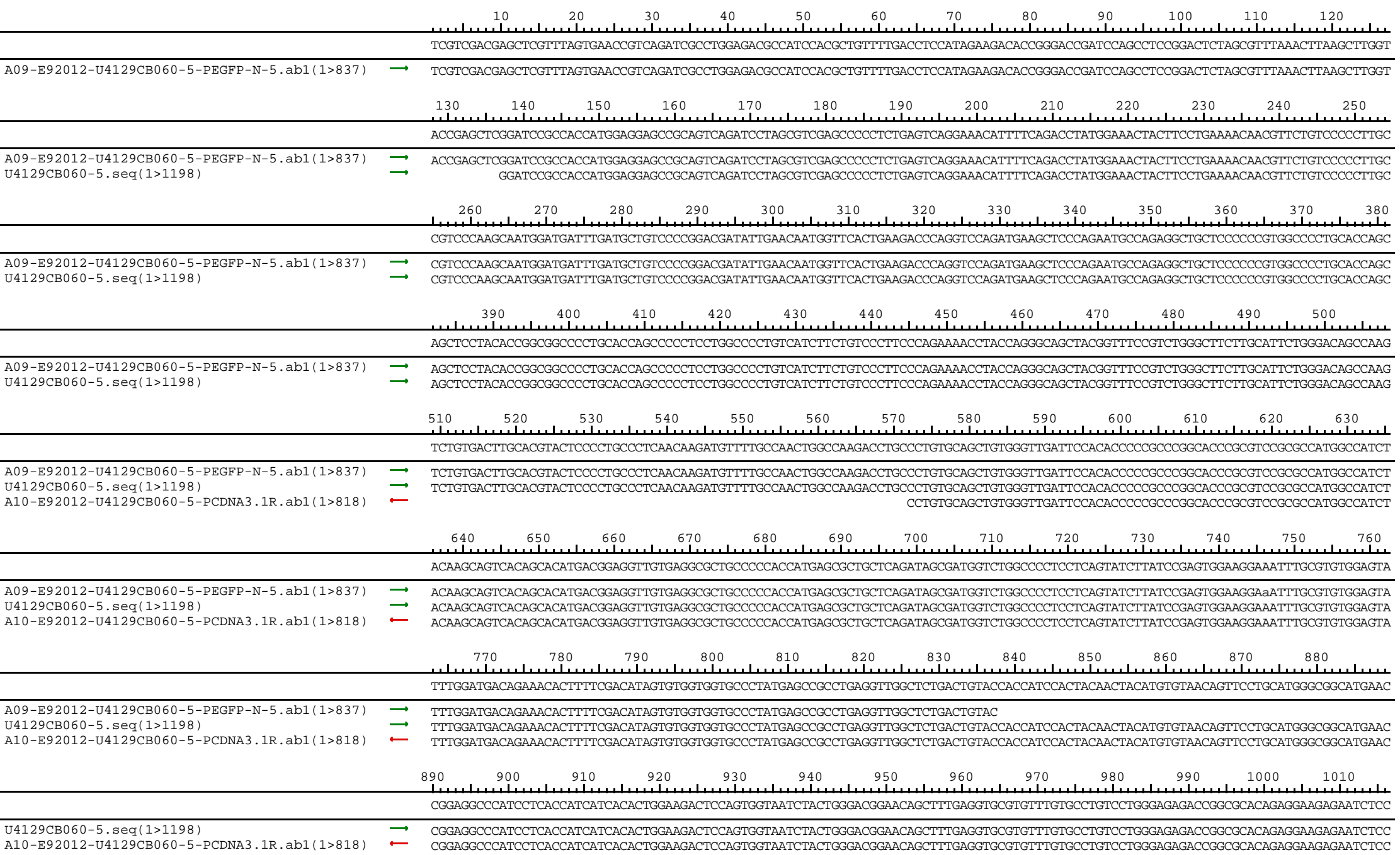

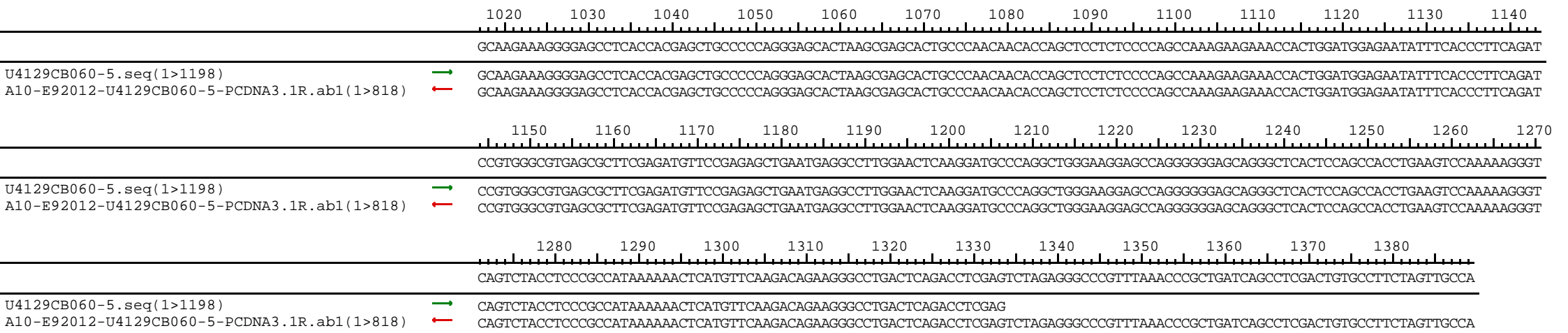

Supplement: Supplementary file 1 [file ijms-21-06751-s001.zip › Supplemental data/Supplementary File 5/U4129CB060-5-sqd.pdf]

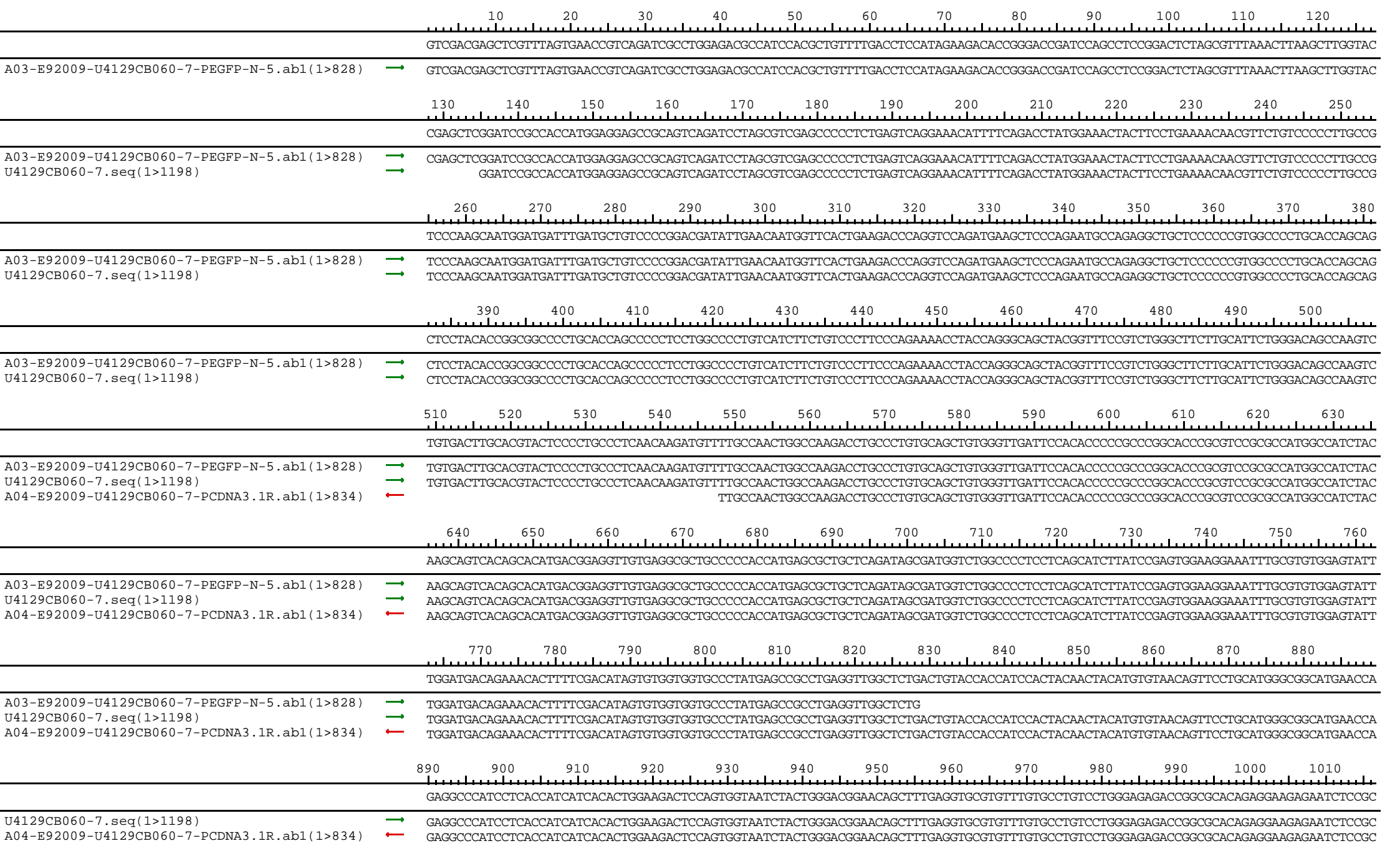

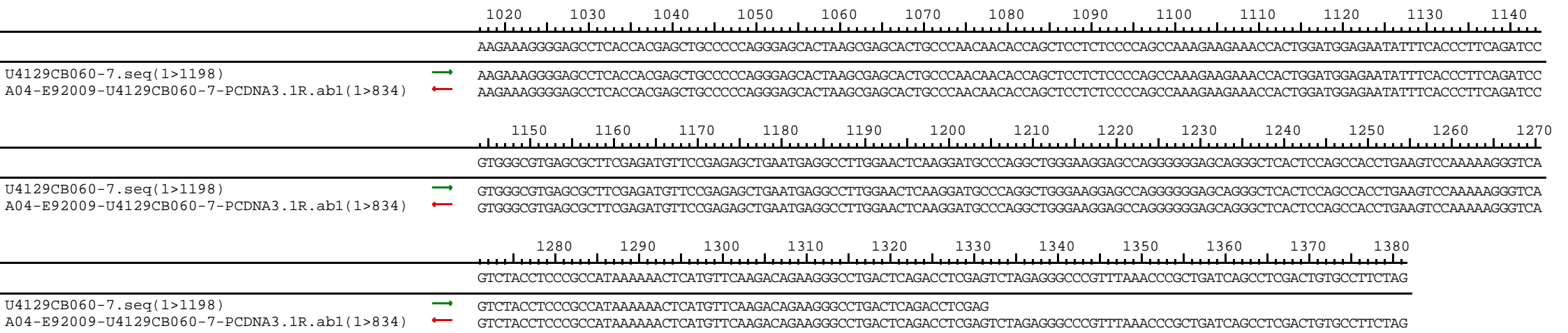

Supplement: Supplementary file 1 [file ijms-21-06751-s001.zip › Supplemental data/Supplementary File 7/U4129CB060-7-sqd.pdf]

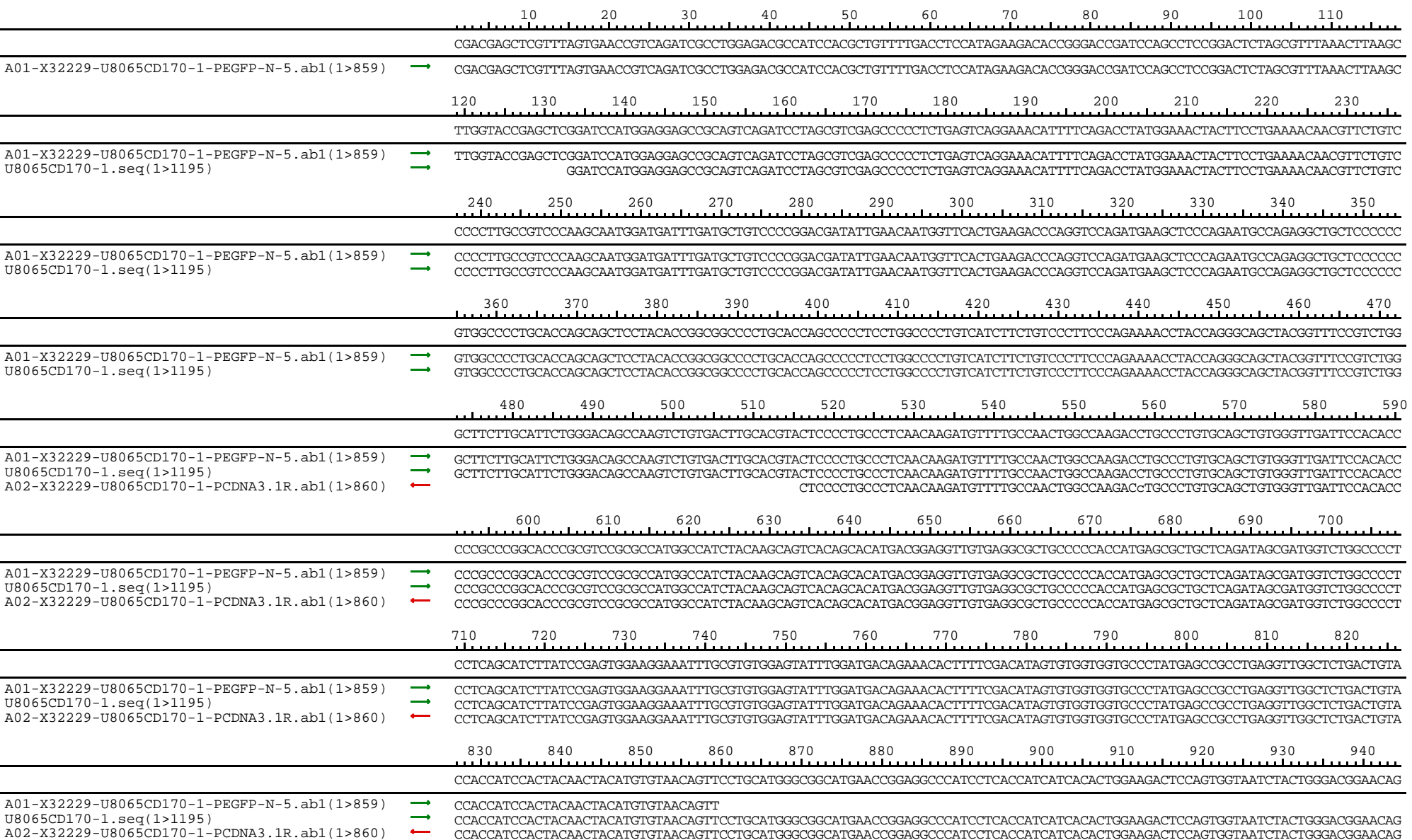

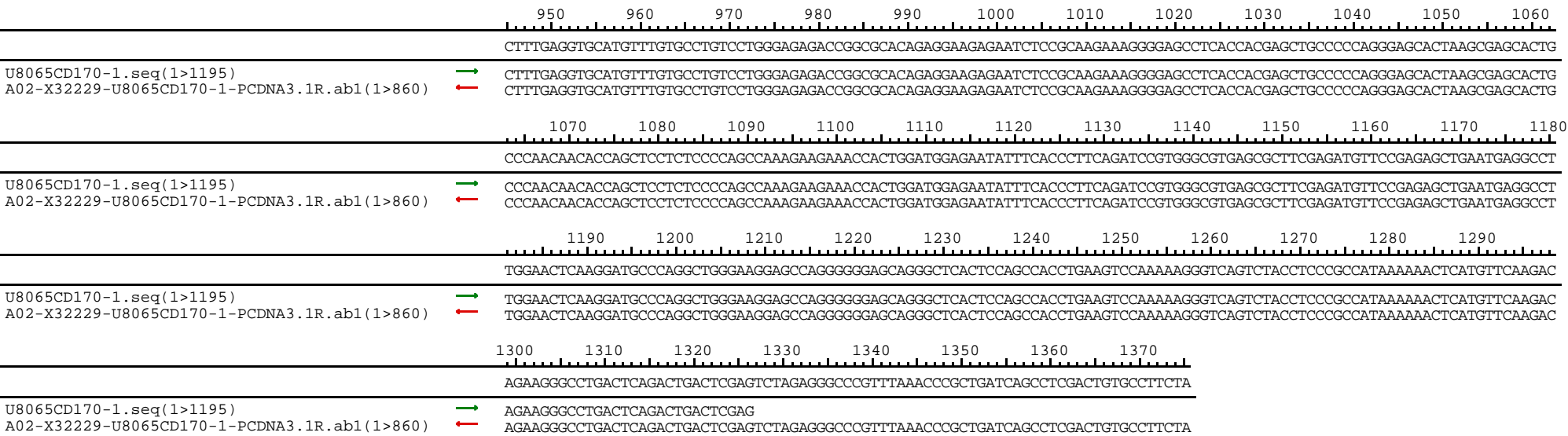

Supplement: Supplementary file 1 [file ijms-21-06751-s001.zip › Supplemental data/Supplementary File 9/U8065CD170-1-sqd.pdf]
